# Undervalued Contribution of OVOCs to Atmospheric Activity: A Case Study in Beijing

**DOI:** 10.3390/toxics14010077

**Published:** 2026-01-14

**Authors:** Kaitao Chen, Ziyan Chen, Fang Yang, Xingru Li, Fangkun Wu

**Affiliations:** 1 Analysis and Testing Center, Department of Chemistry, Capital Normal University, Beijing 100048, China; ckt0106@126.com (K.C.); zy.chen27@foxmail.com (Z.C.); fangyang1107@163.com (F.Y.); lixr@cnu.edu.cn (X.L.); 2 Key Laboratory of Geographic Information Science of the Ministry of Education, School of Geographic Sciences, East China Normal University, Shanghai 200241, China; 3 State Key Laboratory of Atmospheric Boundary Layer Physics and Atmospheric Chemistry (LAPC), Institute of Atmospheric Physics, Chinese Academy of Sciences, Beijing 100029, China

**Keywords:** volatile organic compounds (VOCs), volatile phenols, ozone generation potential (OFP), secondary organic aerosols (SOA), source apportionment

## Abstract

VOCs are significant precursors for the formation of O_3_ and SOA, directly impacting human health. This study employs multiple approaches to analyzing atmospheric VOCs by focusing on OVOCs including aldehydes, ketones, and phenols, with a case study in Beijing, China. We analyzed the concentration levels and compositions of VOCs and their atmospheric activities, offering a new perspective on VOCs. This analysis was conducted through offline measurements of volatile phenols and carbonyl compounds, complemented by online VOC observations during the summer period of high O_3_ levels. The total atmospheric VOCs concentration was found to be 51.29 ± 10.01 ppbv, with phenols contributing the most (38.87 ± 11.57%), followed by carbonyls (34.91 ± 6.85%), and aromatics (2.70 ± 1.03%, each compound is assigned to only one category based on its primary functional group, with no double counting). Carbonyls were the largest contributors to the OFP at 59.03 ± 14.69%, followed by phenols (19.94 ± 4.27%). The contribution of phenols to the SOAFP (43.37 ± 9.53%) and the L_OH_ (67.74 ± 16.72%) is dominant. Among all quantified VOC species, phenol and formaldehyde exhibited the highest species-level contributions to atmospheric reactivity metrics, including L_OH_, OFP and SOAFP, owing to their combination of elevated concentrations and large kinetic or MIR coefficients. Using the PMF model for source analysis, six main sources of volatile organic compounds were identified. Solvent use and organic chemicals production were found to be the primary contributors, accounting for 31.76% of the total VOCs emissions, followed by diesel vehicle exhaust (17.80%) and biogenic sources (15.51%). This study introduces important OVOCs such as phenols, re-evaluates the importance of OVOCs and their role in atmospheric chemical processes, and provides new insights into atmospheric VOCs. These findings are crucial for developing effective air pollution control strategies and improving air quality. This study emphasizes the importance of OVOCs, especially aldehydes and phenols, in the mechanism of summer O_3_ generation.

## 1. Introduction

The acceleration of urbanization and industrialization has led to the emergence of severe ozone (O_3_) and haze pollution as a significant environmental concern in China’s urban atmosphere. This pollution is characterized by the co-existence of O_3_ and fine particulate matter (PM_2.5_), which presents a unique challenge for environmental management [1]. From January to July 2023, the average proportion of “good days” (daily AQI ≤ 100, GB 3095–2012 [2]) in the “2 + 26” cities of the Beijing–Tianjin–Hebei and surrounding regions was 56.6%, showing a year-on-year decline of 5.6%. The average proportion of days with heavy and above pollution was 4.8%, a year-on-year increase of 2.8%. The average concentration of PM_2.5_ was 43.18 ± 12.57 μg/m^3^, a year-on-year flat. The average concentration of O_3_ was 97.2 ± 57.68 ppbv, a year-on-year increase of 2.7% [3]. The data indicate an upward trajectory in O_3_ levels within the Beijing-Tianjin-Hebei region. Volatile Organic Compounds (VOCs) are important precursors of O_3_ and PM_2.5_ [4] as well as precursors of secondary organic aerosols [5]. VOCs are a ubiquitous environmental contaminant, encompassing a diverse array of chemical compounds, including hydrocarbons, oxygenated VOCs, and nitrogenous or sulfurous VOCs. These substances are volatile at room temperature and can enter the atmosphere through a number of pathways, including industrial emissions, transportation, and natural biological processes. VOCs not only have a direct impact on air quality, but also generate ozone and fine particulate matter through photochemical reactions, causing indirect harm to human health and ecosystems [6]. Since around 2017, the composition of atmospheric VOCs in urban areas of China has exhibited a clear shift, with oxygenated VOCs (OVOCs) increasingly replacing alkanes as the dominant chemical class. Consistent with this trend, OVOCs were identified as the predominant component of urban atmospheric VOCs, accounting for 23.76%, 52%, and 61.21% of the total VOC concentrations in Shijiazhuang (2017) [7], Lhasa (2021) [8], and Weihai (2022) [9], respectively. Conversely, alkanes constituted only approximately 20% of the total VOCs, a notable deviation from the findings of the preceding study, which indicated that alkanes were the primary component of VOCs [10,11,12].

However, research on OVOCs has predominantly focused on aldehydes, ketones, organic acids, alcohols, and ethers, while phenolic compounds have received comparatively less attention [13,14,15], and volatile phenols, which are also members of OVOCs, have been neglected for a long time [16]. This may be attributed to their intricate chemical structures, stronger photochemical activities, and technical constraints. Volatile phenols are a highly toxic industrial pollutant, primarily derived from paper, chemical wastewater, ammonia, and other industrial processes. It is also a type of aromatic compound, with a benzene ring that contains at least one hydroxyl group (·OH) [17]. The multiplicity of applications of phenols has resulted in their progressive accumulation in the environment, particularly in the atmosphere, water, and soil. Various higher concentrations of phenols were measured in sewage (up to 53 ppmv), ambient water (1.5~100 ppbv), drinking water (unquantified), groundwater (1.9~10 ppbv), rainwater (0.075~1.2 ppbv), sediment (>10 ppbv) and ambient air (0.03~44 ppbv) [18].

Atmospheric monitoring and mechanism studies commonly report and/or target a broader suite of functionalized phenolics beyond phenol and cresol isomers, including higher alkyl-substituted phenols (e.g., dimethyl- and trimethylphenols), naphthols, and nitrated phenols (nitrophenols). This broader functional coverage is relevant because substitution patterns and nitration chemistry can alter both gas-phase reactivity and SOA formation pathways [19]. It can be observed that the concentration of volatile phenols exhibits considerable variation across different regions. Furthermore, the concentration of volatile phenols was found to be higher in urban areas than in rural areas. With regard to phenol, for instance, the observed concentrations in urban areas were as follows: 0.08 ppbv in Portland [20], 0.11 ppbv in Milan [21], and 0.03 ppbv in Santiago de Chile [22]. The concentration of phenol in a rural area of the UK was observed to range from 0~0.01 ppbv [23], a figure that is considerably lower than that recorded in urban areas. This perspective is corroborated by the findings of Delhomme et al. [24], which indicate that urban concentrations > suburban areas > rural settings.

Volatile phenols are plentiful in the atmosphere and originate from a multitude of sources, including biomass combustion [16,25], vehicle exhaust [26], and other anthropogenic activities [27]. In addition, phenolic species can be produced and further transformed through OH-initiated atmospheric oxidation, in which aromatic hydrocarbons form phenols as first-generation products and phenols/cresols are subsequently oxidized to dihydroxy benzenes such as catechol [28,29,30]. Among the aforementioned sources, combustion processes and vehicle exhaust emissions represent significant primary sources of phenols [31], while the chemical reactions of benzene, toluene, and other aromatic compounds in the presence of ·OH represent important secondary mechanisms for the generation of phenols [32].

In addition to the well-known hydrocarbons, aldehydes, and ketones, atmospheric phenolic compounds also play a pivotal role in urban air pollution events. These compounds can undergo photochemical reactions with ·OH, NO_3_ radicals (·NO_3_), and triplet excited carbonyls (^3^C*), which are formed via photosensitization following intersystem crossing of carbonyl-containing chromophores, to generate photo-oxidized products and secondary organic aerosols in the troposphere, with reported mass yields approaching 100% [16,17,25]. ^3^C* are now recognized as important non-radical oxidants in atmospheric multiphase chemistry, capable of initiating hydrogen atom transfer, electron transfer, and energy transfer reactions with phenolic compounds under actinic irradiation [33].

In the tropospheric gas phase, the dominant sink of phenolic VOCs is radical-initiated oxidation rather than direct ozonolysis. Daytime loss is typically governed by OH addition to the aromatic ring, while nighttime processing can be driven by NO_3_-initiated oxidation and nitration chemistry under NO_X_ [34,35,36]. Cl-atom reactions may be episodically important in chloride-influenced environments. Recommended rate coefficients for phenol and cresol isomers are on the order of 10^−11^–10^−10^ cm^3^ molecule^−1^ s^−1^ for OH, whereas NO_3_ reactions are generally slower but can be atmospherically consequential at night due to elevated [NO_3_] and efficient formation of nitrated products; these evaluated kinetics are available in community-recommended compilations (AtmVOCkin) and IUPAC evaluations [37]. Mechanistically, OH oxidation of phenols proceeds predominantly via OH addition to the aromatic ring to form hydroxy cyclohexadienyl radicals, followed by O_2_ addition and RO_2_ chemistry [28,38,39]. Under urban high-NO conditions, RO_2_–NO reactions efficiently propagate radical chains and favor formation of ring-retaining oxygenated aromatics (e.g., dihydroxy benzenes such as catechol and related quinone-type products), whereas under lower-NO conditions RO_2_ pathways can shift toward peroxy radical autoxidation and formation of highly oxygenated low-volatility products that contribute to SOA [39,40,41].

**Figure 1:**
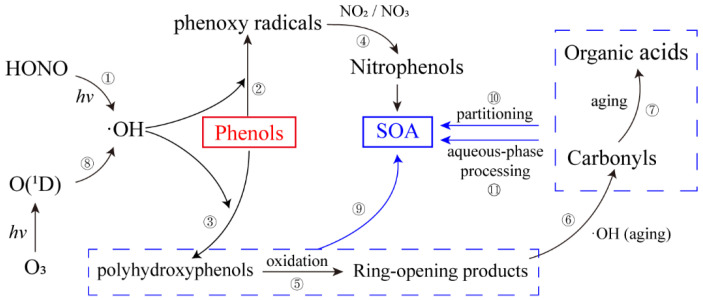
Literature-supported conceptual reaction classes for functionalized phenolics in urban air, covering phenol/cresols, higher alkyl-substituted phenols (dimethyl- and trimethylphenols), and nitrated phenols (nitrophenols), including ring-retaining oxygenation, nitration, and ring-opening oxidation branches. The formation of SOA is indicated at stages where low-volatility oxygenated and nitrated products, including ring-opening products, contribute to particle-phase mass through gas–particle partitioning and/or aqueous-phase processing. Each number corresponds to a reaction class with representative references: ① [45], ② [46,47], ③ [48], ④ (nitration/nitrophenol formation and SOA relevance) [34,43,49], ⑤ (multi-generation oxidation including higher substituted phenols) [42,49,50,51], ⑥ [50,52], ⑦ [53], ⑧ [54,55], ⑨ [35,56], ⑩ [56,57,58], ⑪ [59,60].

Beyond phenol and cresol isomers, kinetic and mechanistic evidence also exists for more highly alkyl-substituted phenols (including trimethylphenols), indicating that the same radical-initiated oxidation framework (dominant OH addition to the aromatic ring followed by RO_2_ chemistry) can be extended across higher substituted phenolic precursors. In parallel, NO_X_-driven nighttime chemistry not only oxidizes phenols but also promotes nitration, yielding nitrated phenols that are atmospherically persistent enough to be observed and are mechanistically relevant to aerosol formation. Importantly, nitrophenol formation and subsequent SOA-relevant evolution have been explicitly demonstrated in laboratory and modeling studies; therefore, both higher substituted alkyl phenols (up to trimethylphenols) and nitrated phenols are explicitly included in our conceptual map (Figure 1) to reflect the functional range of atmospheric phenolics [40,42,43].

These compounds then participate in the broader atmospheric chemical processes. From a gas-phase kinetics perspective, many atmospheric phenolic VOCs (including alkyl-substituted phenols) react efficiently with OH via aromatic-ring addition during daytime, while NO_3_-initiated oxidation coupled with NO_2_-driven nitration at night favors the formation of nitrated phenols that can further contribute to SOA-relevant product evolution (Figure 1) [37,43]. The gas-phase reactions of these compounds and substituted phenols with atmospheric oxidants, including ·OH, ·NO_3_, Cl atoms, and O_3_, represent a primary degradation mechanism, as illustrated in Figure 1. The study of the transformation and degradation reaction mechanism of guaiacol triggered by atmospheric ·OH demonstrated that the products nitroguaiacol, methoxybenzoquinone, hydroxyphenyl formate, 2-methoxybenzene-1,3-diol, and dialdehyde all contributed favorably to the formation of SOA. Additionally, nitroguaiacol was experimentally detected as the main constituent of SOA under conditions of high concentration of NO_2_ during nighttime hours [44]. As demonstrated by Xiao et al. [16], in the typical polluted atmosphere of Beijing in winter, phenols from biomass combustion contribute significantly to the formation of aqueous SOA (aqSOA), and the rate of aqSOA formation can be up to 0.42 μg/(m^3^·h) at midday, which accounts for 15% of the total aqSOA formation rate. Therefore, the monitoring and control of volatile phenols is an important issue in environmental science research and policy making.

Through comprehensive investigation of the field of atmospheric VOCs, we have identified several shortcomings in the current body of research. These include the incomplete nature of fundamental data and the lack of comprehensive atmospheric activity. We employed volatile phenols as a novel approach for in-depth investigation. We conducted atmospheric observations in Beijing, China, and analyzed atmospheric volatile phenols by solid-phase solvent extraction and high-performance liquid chromatography (HPLC) to complete the study of the fundamental data of VOCs. Our objective was to introduce an innovative perspective that could potentially inject new ideas and methods for regional ozone pollution control, expand the research horizons of atmospheric VOCs, particularly OVOCs, and provide theoretical guidance for the future management of regional ozone pollution. It is anticipated that this novel research approach will facilitate the introduction of novel ideas and methodologies into the field of regional ozone pollution control. Furthermore, it is hoped that this research will expand the scope of research into atmospheric VOCs, with a particular focus on OVOCs. Finally, it is expected that this research will provide theoretical guidance for future regional ozone pollution control. Therefore, the present study was conducted from 27 June to 19 July 2023 in Beijing to observe atmospheric volatile phenols as well as alkanes, olefins, aromatics, carbonyl compounds, and other traditional types of VOCs in the external field. The main objectives of this study are (1) to understand the emission characteristics of VOCs based on the volatile phenol perspective, (2) to fill in the missing chemical activity of atmospheric VOCs through the estimation of ·OH depletion rate, ozone generation potential and secondary organic aerosol generation potential, and (3) to identify the source categories of VOCs and to estimate their contributions by using the PMF model.

## 2. Materials and Methods

### 2.1. Sample Collection

Beijing is located in the northern part of the North China Plain, surrounded by mountains in the west, north and northeast, and a plain sloping gently toward the Bohai Sea in the southeast. Beijing has a warm temperate semi-humid and semi-arid monsoon climate, with hot and rainy summers and cold and dry winters. The sampling point of this study is located on the third-floor platform of the experimental building of Beijing Capital Normal University (latitude 22.73° N, longitude 112.93° E; approximately 60 m above ground). The experimental building is situated in the middle of the city, adjacent to the West Third Ring Road, the main transportation artery in Beijing. It is also in close proximity to gas stations, restaurants, and residential areas. The surrounding area is devoid of any discernible sources of pollution, which renders it an apt representation of the typical urban environment in Beijing. This environment is characterised by a convergence of residential, transportation and commercial activities, and exhibits the typical urban characteristics observed in such settings. The summer season is associated with a heightened prevalence of ozone pollution [7]. In this study, we opted to conduct concurrent offline and online observations during the period of elevated ozone levels, specifically from 27 June to 19 July 2023.

The analysis of phenolic compounds is determined in accordance with the methods published by the Ministry of Ecology and Environment of China (https://www.mee.gov.cn/, Last accessed on 1 December 2025). The samples were collected using an atmospheric sampler (Beijing Kangwei Nengte Environmental Technology Co., Ltd., Beijing, China) and XAD-7 resin adsorption columns (inner diameter 6 mm, outer diameter 8 mm, length 11 mm, Beijing Ke’an Labor Protection New Technology Company, Beijing, China). The sampling flow rate was established at 0.3 L/min, with a sampling duration of 4 h. After completion, the column was closed at both ends with PTFE caps, wrapped with aluminum foil, placed in an airtight container, brought back to the laboratory, stored in a refrigerator at 4 °C or below to protect it from light, and analyzed within 14 days. A total of 48 valid samples and 3 blank samples of volatile phenols, 44 valid samples and 2 blank samples of carbonyls were collected in this study. The Ecological Environment Research Center of the Chinese Academy of Sciences provided online data on VOCs, including alkanes, alkenes, and aromatics, in ambient air for the same period.

### 2.2. Sample Analysis

Prior to analysis, the phenolic compounds were subjected to solid-phase extraction. The sampling tubes were brought to room temperature and washed by slowly adding 5 mL of methanol (HPLC Grade, 99.9%, Fisher Chemical, Waltham, MA, USA) from the outgassing end of the sampling tubes using a solid-phase extraction device. The eluent flowed naturally from the outgassing end, and was collected in a 5 mL centrifuge tube and blown down to less than 2 mL using a gentle stream of nitrogen gas. The solution was diluted with methanol to 2 mL and filtered through an organic phase filter membrane (0.22 μm) into a brown liquid phase injection vial for measurement.

Following sample processing, the samples were subjected to qualitative and quantitative analysis via HPLC (Thermo Fisher, Waltham, MA, USA, UltiMate 3000). The chromatographic column utilized was an Acclaim 120 C18 column (5 μm, 25 cm × 4.6 mm) with the column temperature set at 40 °C, the injection volume of 10.0 μL, the detection wavelength of 280 nm, the flow rate of 1.5 mL/min, and the mobile phases of acetonitrile (HPLC Grade, 99.95%, Fisher Chemical) and ultrapure water with 0.1% formic acid (HPLC Grade, 99%, DIKMA, Foothill Ranch, CA, USA) added. The elution gradient is detailed in Supplementary Materials Table S1. Additionally, an additional 5 min equilibration method was established to linearly change the solvent composition from 100% acetonitrile to 30% acetonitrile/70% water before running the next sample.

For further details on the methodology employed in the collection of samples and the analytical techniques used for the analysis of carbonyl compounds, please refer to Supplementary Materials Text S1.

### 2.3. Quality Assurance and Quality Control

The procedures for sample collection and analysis in this study were conducted in accordance with established standards to ensure the integrity and reliability of the data throughout the entire process, from the initial stages of pre-sampling preparation and field collection to the final stages of sample preservation, transportation, analysis, and data processing. The discrepancy in flow rate before and after collection is less than 5%. At least one blank is determined for each batch of samples, and the result is below the method detection limit.

The standard curves of phenols and carbonyls are presented in Supplementary Materials Texts S2 and S3, respectively. The standard curves and the correlation coefficients are shown in Supplementary Materials Tables S2 and S3. The correlation coefficients for the phenols range from 0.9991 to 0.9996, while those for the carbonyls range from 0.997 to 1.000. The method limit of detection (LOD) was calculated using the EPA TO11A method. A standard solution at a concentration close to the desired LOD was prepared and analyzed seven times. The standard deviation was calculated, and the LOD for each compound was determined at the 99% confidence level (see Supplementary Materials Tables S4 and S5). The detection limit for phenols was found to be 0.1538~0.6880 ppbv at a sampling volume of 72 L. Similarly, the detection limit for carbonyls was determined to be 2.5~10.4 × 10^−3^ ppbv at a sampling volume of 144 L.

The relative standard deviations (RSDs) of the carbonyls and phenols were calculated, and the RSD_i of the carbonyls and phenols were found to be less than 2.80% and 7.54%, respectively. Two parallel samples were collected at the same place at the same time, and the relative deviations of each substance were calculated to evaluate the precision of the method, and the RSD_m of carbonyl compounds and phenolic compounds were lower than 3.63% and 7.51%, respectively. The spiked recoveries of carbonyls and phenols exhibited a range of 62.17% to 111.70% and 96.65% to 126.22%, respectively, with the associated errors falling within the acceptable range. The results demonstrate that the method is highly reliable and can be employed in subsequent studies. The detailed evaluation results are presented in Supplementary Materials Tables S4 and S5.

### 2.4. Atmospheric Chemical Activity Analysis

#### 2.4.1. OH Radical Loss Rate Calculation Method (L_OH_)

OH radicals have strong oxidizing properties and are capable of reacting with most pollutants in the atmosphere, thus affecting the atmospheric chemical lifetimes of pollutants [61].

During night, the altitude of the atmospheric boundary layer and the concentration of OH radicals are low, which prevents the removal of atmospheric pollutants by atmospheric diffusion or ·OH degradation, leading to their accumulation near the surface and increasing atmospheric reactivity. During the daytime, the dispersion of air pollutants increases as the height of the mixed layer rises. Concurrently, an augmentation in the concentration of OH radicals augments the capacity for pollutant elimination, thereby curbing the activity of pollutants. Consequently, the activity of pollutants can be quantified by calculating the ·OH loss rate (L_OH_), which enables the contribution of each substance to the atmospheric reaction to be determined. The specific calculation formula is as follows [62]:

(1) $$L_{OH}=\frac{R}{\left[\cdot OH\right]}=k_{OH}\times c$$

(2) $$L_{OH}=\sum c_{i}\times k_{OH,i}=K_{total}\times c_{total}$$

(3) $$R=k_{OH}\times c\times [\cdot OH]$$

where R is the reaction rate; [·OH] is the volume fraction of OH radicals; $k_{OH,i}$ is the reaction rate constant between pollutant i and OH radicals. All K_OH_ values are taken from the latest dataset provided by McGillen, M. R. et al. (AtmVOCkin-3.1) [37]; c is the volume fraction of a particular pollutant; $c_{i}$ and $c_{total}$ are the volume fraction of pollutant i and the total volume fraction of pollutants, respectively; $k_{total}$ is the total reaction rate constant.

#### 2.4.2. Ozone Formation Potential (OFP)

The maximum ozone formation potential (OFP) of a pollutant can be estimated from its maximum incremental reactivity (MIR) value. This approach allows for the quantification of the maximum contribution of atmospheric pollutants to ozone production. Furthermore, the magnitude of the OFP values allows for the identification of the pollutant species that contribute the most to the production of O_3_. Additionally, the sum of the OFP values of all the species can be used as a relative representation of the overall activity of air pollutants in the region. The specific calculation formula is as follows [63]:

(4) $${OFP}_{i}={MIR}_{i}\times c_{i}$$

where ${OFP}_{i}$ is the maximum ozone production potential of pollutant I, ${MIR}_{i}$ is the maximum incremental reactivity of pollutant I, the value of which is adopted from [64,65]; and $c_{i}$ is the volume fraction of pollutant i.

#### 2.4.3. Secondary Organic Aerosol Formation Potential (SOAFP)

SOA represents the most abundant component of organic aerosol, which can be generated through the photochemical oxidation of VOCs [66]. The secondary organic aerosol formation potential (SOAFP) is a common method for characterizing the contribution of SOA generation from VOCs [67]. The most widely used approach is the fractional aerosol coefficient (FAC) [68]. The specific calculation formula is as follows:

(5) $${SOAFP}_{i}={FAC}_{i}\times c_{i}$$

where SOAFP_i_ is the SOA formation potential; FAC_i_ is the fraction aerosol coefficient of species i. The FAC value is adopted from refs. [69,70,71] and ref. [72]; $c_{i}$ is the emission concentration of VOCs of species i.

## 3. Results and Discussion

### 3.1. Emission Characteristics

**Figure 2:**
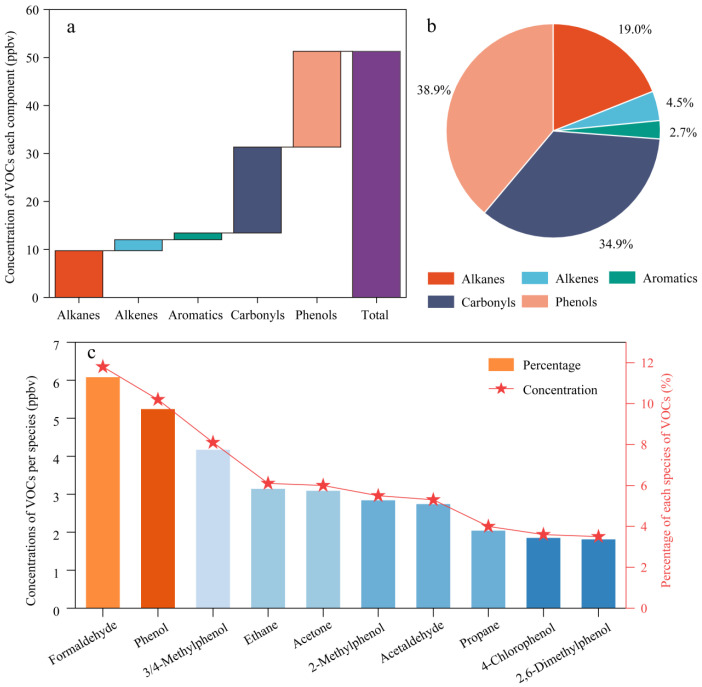
Concentration values (**a**), percent contribution (**b**), and top 10 VOC species (**c**) for each component of VOCs.

A total of 79 VOC species were obtained from this study, including 28 alkanes, 9 alkenes, 15 aromatics, 17 carbonyls, 10 volatile phenols, and the specific components are shown in Supplementary Materials Table S6. It should be noted that each compound is assigned to a single category based on its dominant functional group for reactivity analysis. For example, volatile phenolic compounds are counted only as phenols and are not included within the aromatic group. Therefore, direct comparisons of VOC class dominance between this study and NMHC-focused datasets should be interpreted with caution, as differences in species coverage and classification schemes can substantially alter the apparent contribution of individual VOC classes. Figure 2 illustrates the concentration levels of different components of VOCs, the percentage contribution, and the top ten VOC species. From Figure 2a, it can be seen that the total concentration of atmospheric VOCs is 51.29 ± 10.01 ppbv, and the concentration levels of different components are in the order of volatile phenols (19.95 ± 4.50 ppbv) > carbonyls (17.90 ± 6.85 ppbv) > alkanes (9.74 ± 4.05 ppbv) > alkenes (2.29 ± 2.27 ppbv) > aromatics (1.41 ± 1.17 ppbv). As illustrated in Figure 2b, the largest proportion of volatile phenols was observed, accounting for 38.87 ± 11.57% of the total, followed by carbonyls with 34.91 ± 6.85%, and aromatics exhibited the smallest component, at 2.70 ± 1.03%.

We compared results with summer-only observational datasets that explicitly reported the measurement window. A PTR-MS study conducted in Beijing during summer 2018 reported a total VOC mixing ratio of 39.4 ppb for the targeted VOC suite, with OVOCs contributing the dominant fraction [73]. In eastern China, a 1-year GC-based dataset in urban Nanjing reported a summer minimum of 18.5 ± 14.6 ppbv [74], whereas a long-term investigation in an industrial area of Nanjing reported a summer TVOC level of 38.8 ± 10.2 ppbv [4]. In western China, a petrochemical-industrial campaign in Lanzhou during summer 2019 reported a TVOC level of 50.8 ± 46.1 ppbv [75], comparable to the present study in magnitude. In the Sichuan Basin, an urban campaign in Chengdu during summer (June–August 2019) reported an average VOC concentration of 36.63 ± 12.92 ppbv [76]. Collectively, these summer-only comparisons place our observed TVOC (51.29 ppbv) at the higher end of summer urban datasets, while the consistently high OVOC/oxygenated fraction reported for Beijing summer observations support the robustness of OVOC dominance in photochemically active periods.

Nevertheless, volatile phenols and carbonyls together contributed more than 70% of TVOCs in this study, highlighting that OVOCs (including oxygenated aromatics when measured) can dominate summer VOC composition in Beijing. This also indicates that comparisons based solely on NMHC-focused species lists may systematically under-represent oxygenated aromatics and thereby bias the inferred “dominant” VOC classes in summertime megacities.

Building on this OVOC-dominated profile, the compositional contrast among VOC classes provides mechanistic insight into the summertime atmospheric processing regime in urban Beijing. The exceptionally large phenolic fraction, together with the substantial carbonyl loading, indicates that oxygenated products are not merely secondary or marginal components but constitute a major reservoir of ambient VOC carbon during summer. Such a pattern is characteristic of an environment with elevated temperatures and strong solar radiation, where photochemical oxidation of primary hydrocarbons proceeds efficiently and continuously converts parent compounds into oxygenated products, while volatilization from surfaces and materials further enhances the abundance of oxygenated aromatics [77]. Meanwhile, the very small fraction of non-oxygenated aromatics does not necessarily imply a weak influence of aromatic precursors. Instead, it likely reflects rapid daytime oxidation that limits the atmospheric persistence of parent aromatic hydrocarbons and shifts the chemical burden toward their oxygenated derivatives, which are classified as OVOCs rather than conventional NMHC aromatics [78].

In addition, the distribution of the top ten VOC species demonstrates a pronounced concentration centralization, whereby a limited number of compounds account for a large fraction of the total mixing ratio. The co-occurrence of phenol and methylphenols with formaldehyde and acetone among the most abundant species suggests that both secondary formation and continuous source replenishment sustain these compounds at elevated levels [79]. This concentration structure implies that source signatures and transformation pathways converge on a few chemically stable or persistently produced species, allowing them to dominate bulk concentration statistics even in the presence of a chemically diverse VOC mixture. Furthermore, the relatively large standard deviations observed for phenols and carbonyls indicate strong temporal variability, which is consistent with the combined influence of boundary-layer evolution, episodic emissions, and short-lived photochemical production events [80]. Overall, these features highlight that summertime VOC composition in Beijing is strongly shaped by oxygenated compounds and underscore that hydrocarbon-focused measurement strategies may substantially underestimate the contribution of oxygenated aromatics unless volatile phenols and related OVOCs are explicitly included.

### 3.2. Photochemical Reactivity

**Figure 3:**
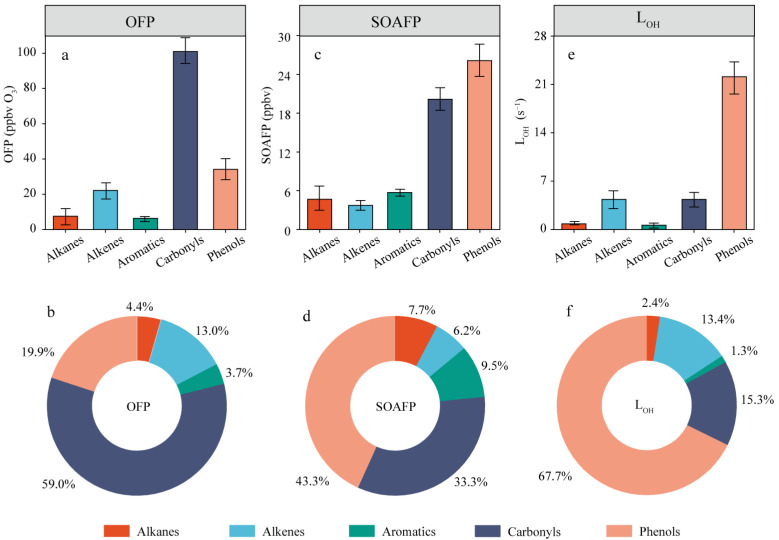
Calculated chemical reactivity values for each component of VOCs. (**a**,**b**) denote the maximum ozone production potential (OFP) and its percentage contribution for each component, respectively; (**c**,**d**) denote the secondary organic aerosol production potential (SOAFP) and its percentage contribution for each component, respectively; and (**e**,**f**) denote the rate of OH radical depletion (L_OH_) and its percentage contribution for each component, respectively.

In the current literature, estimates of the atmospheric chemical activity of VOCs are primarily based on three key parameters: the maximum ozone production potential (OFP), the secondary organic aerosol production potential (SOAFP), and the rate of loss of ·OH (L_OH_). Therefore, the present study explores these three activity calculations separately, and the estimation results are shown in Figure 3.

#### 3.2.1. OFP

The total ozone formation potential for VOCs is 171.19 ± 76.55 ppbv O_3_. The OFP values, as well as the contribution percentages of each VOC component, are shown in Figure 3a,b, respectively. Carbonyl compounds collectively accounted for the largest fraction of the total OFP (100.99 ± 25.15 ppbv, 59.03 ± 14.69%), primarily driven by a small number of highly reactive carbonyl species (e.g., formaldehyde and acetaldehyde) with high MIR values and elevated abundances. This phenomenon reflects secondary oxidation and fragmentation pathways under Beijing’s intense summer photochemistry: formaldehyde (5.08 ± 1.15 ppbv) and acetaldehyde (2.27 ± 0.70 ppbv) are already at high abundance levels, while small-molecule carbonyls such as glyoxal/glyoxalate are also significantly present. This indicates that alkenes/isoprene and aromatic systems rapidly ‘converge’ towards small-molecule carbonyls under high NO conditions. This shifts the OFP budget towards a ‘secondary-product-dominant’ urban summer pattern, an ‘aldehyde-dominated OFP’ configuration repeatedly observed in summer studies across other cities. Followed by volatile phenols, with an OFP value of 34.15 ± 7.31 ppbv O_3_, accounting for 19.94 ± 4.27% of the total; and aromatics had the lowest OFP value of 6.35 ± 2.18 ppbv O_3_, accounting for 3.70 ± 1.27% of the total. Compared to alkanes, alkenes and aromatics contribute more to O_3_ formation due to their high reactivity, although their concentrations are lower than those of alkanes, and the results are consistent with [81]. The dominant species were formaldehyde, acetaldehyde, phenol, isoprene and methylglyoxal. Among them, four aldehydes and ketones, two alkenes and four volatile phenols contributed 55.27 ± 7.69%, 9.90 ± 3.31% and 19.95 ± 6.22% to the OFP, respectively. The sum of the ozone-forming dominant species’ OFPs is 145.70 ± 29.43 ppbv O_3_, representing 85.11 ± 17.19% of the total. This further indicates that the ‘reactivity weighting’ of O_3_ precursors is highly concentrated in a few species and their upstream precursors. Therefore, control strategies should prioritize reducing direct emissions and secondary formation precursors (highly reactive alkenes and isoprene) of carbonyls such as formaldehyde/acetaldehyde, while simultaneously constraining aromatic emission sources capable of generating phenols. By contrast, merely reducing low-reactivity alkanes based on concentration is likely to yield lower ozone emission reduction benefits per unit effort.

#### 3.2.2. SOAFP

The total estimated secondary organic aerosol formation potential for VOCs is 60.44 ppbv. Figure 3c,d shows the SOAFP values and their contribution to each VOC component, respectively. The SOAFP values and contributions of the components were alkanes (4.68 ± 1.47 ppbv, 7.74 ± 2.43%), alkenes (3.74 ± 0.38 ppbv, 6.18 ± 0.62%), aromatics (5.73 ± 0.66 ppbv, 9.48 ± 1.09%), carbonyls (20.15 ± 6.02 ppbv, 33.33 ± 9.96%), and volatile phenols (26.14 ± 3.61 ppbv, 43.25 ± 5.97%). At the class-aggregated level, volatile phenols showed the largest contribution to SOAFP; however, this dominance arises from the high SOA formation potentials of several individual phenolic species (e.g., phenol and methyl phenols), rather than implying uniformly high SOA yields across the entire phenolic class. The ten substances with the highest contribution to SOA include phenol, benzaldehyde, formaldehyde, isoprene, n-dodecane, toluene, acetaldehyde, p/m-xylene, acetone, and cis-2-pentene. Four carbonyls, one alkane, two alkenes, two aromatics and one volatile phenol contributed 33.15 ± 4.97%, 3.83 ± 0.76%, 5.57 ± 0.99%, 5.02 ± 0.60% and 43.37 ± 9.53% to the SOAFP, respectively. The sum of SOAFPs for the top ten SOA-forming dominant species was 54.90 ± 7.98 ppbv, representing 90.83 ± 13.20% of the total.

#### 3.2.3. L_OH_

As shown in Figure 3e, the ΣL_OH_ of the measured VOCs observed in Beijing during the summer of 2023 reached 31.7 s^−1^, which is substantially higher than the <10 s^−1^ level typically reported for global clean regions, and approximately 2.8 times that of the summer “VOC reactivity” in Beijing in 2017 (11.2 s^−1^) [82]. Interregional comparisons further indicate that summertime VOC-related OH sinks in Beijing fall within the magnitude characteristic of “high-reactivity cities.” The average total ·OH reactivity observed in London during summer was 18.1 s^−1^ [83]; In the Pearl River Delta (Backgarden/PRD), summertime diurnal variations showed a midday minimum of approximately 20 s^−1^ and an early-morning peak reaching up to 50 s^−1^ [84]; In Mexico City, summertime daytime OH reactivity averaged around 25 s^−1^ and could surge to ~120 s^−1^ during the morning rush hour [85]; In a suburban forested area near Tokyo, the summertime mean OH reactivity was reported as 11.4 s^−1^ [86]. Together, these summertime observations indicate that the ΣLOH derived in this study not only exceeds background levels typical of temperate cities and the mean values of some metropolitan areas, but also lies within the characteristic range of summer conditions in cities with high emissions and strong photochemical activity.

Phenolic compounds accounted for 67.74 ± 16.72% of ΣLOH during the observation period, exceeding the aggregated contributions of alkenes and carbonyls. This result indicates that, at the class-aggregated level, phenolic compounds constitute an important contributor to OH reactivity in the studied urban atmosphere (Figure 3f). This finding contrasts markedly with earlier conclusions that summertime VOC reactivity in Beijing is typically dominated by alkenes [87,88]. One key reason for this discrepancy is that most previous field campaigns did not systematically include volatile phenols, focusing instead on traditional NMHCs (e.g., C_2_–C_10_ alkanes and alkenes) [89]. Owing to their molecular structures, which combine aromatic π systems with strong electron-donating substituents, phenolic compounds exhibit K_OH_ values substantially higher than those of alkenes with the same carbon number and monocyclic aromatics [62]. Consequently, even at moderate concentrations, phenols can contribute disproportionately to ·OH consumption. Their omission therefore leads to a systematic underestimation of the true summertime ·OH reactivity in urban environments and exaggerates the apparent dominance of alkenes. By incorporating volatile phenols at the species level, the reactivity pattern revealed in this study more closely represents the middle-to-late stages of aromatic hydrocarbon oxidation toward oxygenated aromatic products, pointing to a more advanced degree of secondary oxygenation and a chemical age structure closer to the “aromatic–oxygenated aromatic” transition regime [19].

C_6_–C_10_ species contributed 73.74 ± 2.79% of ΣL_OH_, approximately 3.3 times that of C_2_–C_5_ species, demonstrating that intermediate-carbon-number organics play a dominant role in the OH reactivity budget in urban environments [62]. This feature reflects the tendency of such compounds, under multi-source emission backgrounds (including solvent use, combustion emissions, and secondary formation), to simultaneously exhibit relatively high atmospheric abundances and large K_OH_ values, thereby occupying a substantial fraction of the ·OH sink. It should be noted that OH reactivity is highly species-specific rather than class-specific: the top 5% of high-L_OH_ species comprised only four compounds, yet accounted for 54.27 ± 17.40% of the total L_OH_. These species primarily included 1-naphthol, methyl phenol isomers, phenol, and isoprene, all of which share conjugated π systems combined with electron-donating substituents or high-electron-density double bonds. Under urban high-NO conditions, compounds dominated by ·OH addition reactions more readily promote RO_2_–NO reaction pathways [90,91], leading to a strong concentration of ·OH consumption among a small number of highly reactive species and amplifying the influence of ·OH reactivity on ozone formation. Mechanistically, alkanes primarily undergo H-abstraction reactions, with reaction rates constrained by steric effects and C–H bond dissociation energies, resulting in relatively low unit-concentration contributions to the OH sink [92,93]. In contrast, alkenes and aromatic systems predominantly react via ·OH addition, where substituent electronic effects enhance the branching ratio of addition pathways and accelerate the conversion of RO_2_ to NO reaction channels [92,94], allowing ·OH consumption to participate more directly in ozone formation. Phenolic compounds occupy a critical position within the aromatic oxidation system, as they can originate from both primary emissions and early- or mid-generation products of aromatic hydrocarbon oxidation [95]. Their substantial contribution to the ·OH sink indicates that summertime ozone and secondary organic aerosol formation in Beijing are more likely governed by the combined influence of aromatic hydrocarbons and their oxygenated derivatives, rather than being driven solely by traditional alkene precursors [49,96].

### 3.3. Source Apportionment

In this study, the PMF model was employed to ascertain the six source factors of VOC in Beijing during the summer season. These included fuel evaporation sources, gasoline vehicle exhaust sources, biological sources, diesel vehicle exhaust sources, solvent use and organic chemical sources, and petrochemical industry sources. A detailed textual description of the PMF model can be found in Supplementary Materials Text S4. The results of the PMF model analysis are presented in Supplementary Materials Figure S1.

Factor 1 is dominated by C_6_~C_7_ alkanes, including 2-methylpentane and 3-methylpentane, as well as cis-2-pentene. These species are characteristic of oil and gas evaporation [97,98], and thus factor 1 is regarded as a fuel evaporation source.

Factor 2 is notable for its high content of propylene, trans-2-butene, and cis-2-butene, as well as the presence of benzenes such as p-/m-ethyltoluene, which are the main components of gasoline vehicle exhaust [99,100]. Consequently, this factor was identified as a source of gasoline vehicle exhaust emissions.

Factor 3 is regarded as a biogenic emission source, exhibiting a distinctive chemical signature characterised by isoprene, which serves as an invaluable tracer for identifying vegetative emission sources [101].

Factor 4 contains a high level of n-dodecane, and diesel vehicle exhaust typically employs long-chain alkanes as markers, including n-undecane and n-dodecane [102]. Therefore, factor 4 is regarded as a contributor to diesel exhaust emissions.

Factor 5 contains a variety of OVOCs, including acetone, which is a common solvent. Additionally, solvent use contributes to the formation of benzaldehyde, acetaldehyde, and phenol [103,104]. Furthermore, the substance contains elevated concentrations of phenolic compounds, predominantly derived from the organic chemical industry. Phenol, chlorophenol, naphthol, etc., are extensively utilized in a multitude of organic chemical intermediates and raw materials [105,106,107]. Accordingly, factor 5 was identified as solvent use and organic chemical sources.

Long-chain alkanes with a high proportion of factor 6 are a dominant presence in the petrochemical industry, particularly n-heptane and methylcyclohexane [108]. Thus, this factor was identified as a potential source within the petrochemical industry.

**Figure 4:**
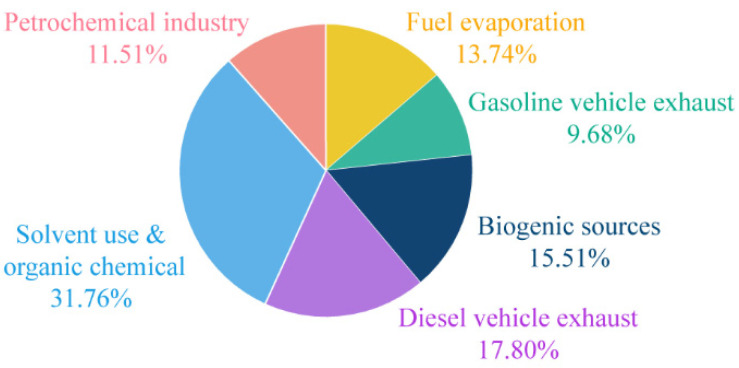
Percentage of source contributions of VOCs in Beijing.

Figure 4 illustrates the relative proportions of the contribution of atmospheric VOCs in Beijing by each source category. The contributions of each source category to VOCs are, in descending order, solvent use and organic chemical sources, diesel use sources, biological sources, fuel evaporation, petrochemical industry, and gasoline vehicle exhaust. It is important to note that solvent use and organic chemical sources (31.76%), as well as motor vehicle exhaust (27.48%, including vehicle exhaust and diesel use), contribute significantly to VOC pollution in Beijing.

**Figure 5:**
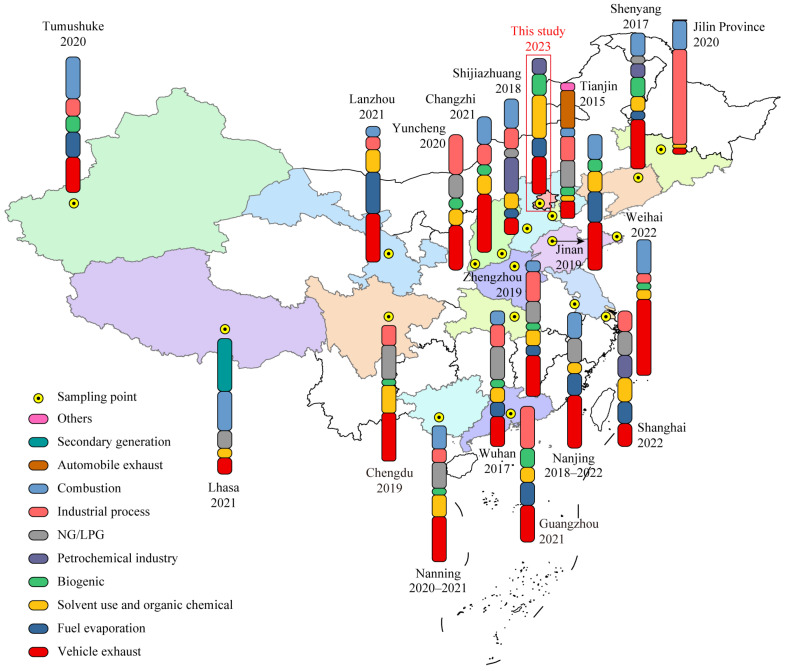
Source profiles of VOCs in 1 province and 18 cities in China. The 1 province and 18 cities included are Jilin Province [111], Beijing (this study), Tianjin [11], Weihai [9], Jinan [112], Shanghai [113], Nanjing [4], Wuhan [114], Nanning [115], Chengdu [116], Lhasa [8], Tumushuke [117], Lanzhou [118], Yuncheng [119], Changzhi [120], Zhengzhou [121], Shijiazhuang [122], Shenyang [123], and Guangzhou [124]. Specific abundances are detailed in Supplementary Materials Table S7.

The results of the atmospheric VOCs source analysis for 1 province and 18 cities in China for the period 2015–2023 are shown in Figure 5. It is possible that the source of VOCs varies from region to region due to different topography, industrial structure, etc. A comparison of the results of PMF source analysis in Beijing with those of 18 other sites in China revealed that vehicle exhaust (including gasoline and diesel exhaust) is the primary source of VOCs in Chinese cities, with a share ranging from 17% to 56% in the majority of cities. Among the aforementioned cities, the contribution of vehicle exhaust to Weihai is the largest (56%); the contribution to Lhasa, Tianjin, and Shijiazhuang is approximately 12%, and the lowest is in Jilin Province (4.6%), which indicates that the concentration of VOCs in urban areas is significantly influenced by vehicles. Secondly, Chinese cities are also heavily influenced by combustion sources, including biomass and coal combustion. The prevalence of the condition in Lhasa is approximately 29.0%, which is comparable to that observed in Tumushuke (30.9%) and higher than that observed in Weihai (25.1%), Shijiazhuang (21.5%), Jilin Province (21.4%), Changzhi (20.3%), and other sites. The diel profile (Supplementary Materials Figure S2) suggested that biomass burning persists in the suburbs of Lhasa [109], despite the suppression of older aerosol sources, such as wood, agricultural residues, cow dung cake burning, and garbage incineration, since the release of the Air Pollution Prevention and Control Action Plan (APAP) in 2013 [8]. Furthermore, incense burning represents another significant source of VOCs in Lhasa [110]. Industrial processes can also significantly affect the concentration of atmospheric VOCs. Especially in Jilin Province, as one of the typical representatives of China’s old industrial bases, with intensive chemical and petrochemical industries, the contribution to VOCs reaches an alarming 70.6%. Solvent use, NG/LPG, biogenic and fuel evaporation are also not negligible for the emission of VOCs in the urban atmosphere, accounting for about 2.73% to 31.76%. In addition, the petrochemical industry plays a significant role in certain urban areas, representing the largest source of VOCs in Shijiazhuang (26.2%), a figure that is higher than that observed in Beijing (11.5%) and Shanghai (16.6%). In addition, unlike other cities in China, the largest source of VOCs in Lhasa is secondary generation (39.0%), which is higher than combustion sources (29.0%) and much larger than NG/LPG (13.0%), vehicle exhaust (12.0%) and solvent use (7.0%). This phenomenon can be mainly attributed to the unique geographical situation of Lhasa, the Tibetan Plateau (Supplementary Materials Figure S3). It is well known that the Tibetan Plateau, with an average altitude of over 4000 m above sea level, has thin air and strong solar radiation. This results in the formation of VOCs being more strongly influenced by solar factors. The typical daytime peaks of OVOCs support this effect, as shown in Supplementary Materials Figure S4 [8]. In summary, by comparing with other sites in China, we found that the sources of VOCs are diverse and complex, especially affected by vehicle exhaust, and the sources of VOCs in Beijing could not escape the results of this analysis.

## 4. Conclusions

This study characterized the concentration levels and compositional features of VOCs and their contributions to atmospheric chemical processes based on summer observations in downtown Beijing. Phenols and carbonyls were identified as the dominant VOC groups, accounting for 38.87% and 34.91% of total concentrations, respectively, highlighting the importance of OVOCs in shaping urban TVOC burdens.

Phenolic species showed strong species-level contributions to SOA formation and OH radical loss, and, when aggregated, ranked second to carbonyl compounds in their contribution to O_3_ formation. Among them, phenol and cresols (including their isomers) were the most reactive species, indicating that volatile phenols are key drivers of urban oxidizing capacity as well as O_3_ and SOA formation.

Fuel evaporation, gasoline vehicle exhaust, biogenic emissions, diesel vehicle exhaust, solvent use and organic chemicals, and the petrochemical industry. Solvent-related and vehicle-related sources were the dominant contributors to VOC pollution in Beijing, providing important implications for regional emission control strategies.

Despite clear evidence for the critical role of OVOCs, several knowledge gaps remain. Multi-season and multi-site observations are needed to better constrain the spatiotemporal variability of phenols and carbonyls and to distinguish primary emissions from secondary formation. Higher time-resolution VOC measurements combined with constraints on radical chemistry would enable more mechanistic attribution of O_3_ and SOA formation. In addition, integrating receptor-based source apportionment with chemical transport modelling is necessary to evaluate sector-specific control strategies under different O_3_ sensitivity regimes. Finally, SOA formation estimates for phenolic compounds should incorporate Beijing-specific NO_X_ levels, aerosol loading, and multiphase chemistry to reduce uncertainties in translating formation potentials into policy-relevant outcomes.

Collectively, these findings strengthen the understanding of VOC pollution in Beijing and provide a clearer basis for targeted air quality management, while also defining priorities for future OVOC-focused observational and modelling studies in megacities.

## Data Availability

The original contributions presented in this study are included in the article/Supplementary Materials. Further inquiries can be directed to the corresponding author.

## Supplementary Materials

The following supporting information can be downloaded at: https://www.mdpi.com/article/10.3390/toxics14010077/s1. Text S1: Sample collection and analytical methods for carbonyl compounds. Text S2: Establishment of standard curve for carbonyl compounds. Text S3: Establishment of standard curve for volatile phenols. Text S4: PMF model. Table S1: HPLC mobile phase elution gradients. Table S2: Standard curves and correlation coefficients for carbonyl compounds. Table S3: Standard curves and correlation coefficients for volatile phenols. Table S4: Method detection limit, precision and recovery rate of labeling for carbonyl compounds. Table S5: Method detection limit (MDL), precision and recovery rate of labeling for volatile phenols. Table S6: Atmospheric VOCs species fractions collected simultaneously. Table S7: Source profiles of VOCs and their contribution percentages in 19 province and cities in China. Figure S1. Source profiles of resolved factors for the PMF model (gray bars represent mixing ratios, red dots represent percentages). Figure S2. Ternary diagram of benzene, toluene, and ethylbenzene. Figure S3. Chinese elevation map. Figure S4. Diel profiles of six decomposed factors (**a**) and concentration diel profiles of key tracers of the Secondary generation source (**b**). References [4,8,9,11,109,111,113,114,115,116,117,120,121,122,123,124,125,126,127,128] are cited in the Supplementary Materials.

## References

[1] Zhang H., Jiang H., Jian G., Li H. (2022). Formation Mechanism and Management Strategy of Cooperative Control of PM_2.5_ and O_3_. Res. Environ. Sci..

[2] (2012). Ambient Air Quality Standards.

[3] Ministry of Ecology and Environment of the People’s Republic of China Ministry of Ecology and Environment Reports on National Ambient Air Quality Conditions in July and January–July. https://www.mee.gov.cn/hjzl/sthjzk/zghjzkgb/.

[4] Mozaffar A., Zhang Y.-L., Lin Y.-C., Xie F., Fan M.-Y., Cao F. (2021). Measurement report: High contributions of halocarbon and aromatic compounds to atmospheric volatile organic compounds in an industrial area. Atmos. Chem. Phys..

[5] Song M.D., Tan Q.W., Feng M., Qu Y., Liu X.G., An J.L., Zhang Y.H. (2018). Source Apportionment and Secondary Transformation of Atmospheric Nonmethane Hydrocarbons in Chengdu, Southwest China. J. Geophys. Res. Atmos..

[6] Ji Y.M., Shi Q.J., Li Y.X., An T.C., Zheng J., Peng J.F., Gao Y.P., Chen J.Y., Li G.Y., Wang Y. (2020). Carbenium ion-mediated oligomerization of methylglyoxal for secondary organic aerosol formation. Proc. Natl. Acad. Sci. USA.

[7] Wang S., Cui J., Feng Y., Liu D., Chen J., Wang M., Wang X., Wang T. (2020). Characteristics and Source Apportionment of VOCs and O_3_ in Shijiazhuang. Environ. Sci..

[8] Ye C., Guo S., Lin W., Tian F., Wang J., Zhang C., Chi S., Chen Y., Zhang Y., Zeng L. (2023). Measurement report: Source apportionment and environmental impacts of volatile organic compounds (VOCs) in Lhasa, a highland city in China. Atmos. Chem. Phys..

[9] Zhang X., Wang L., Jin X., Zhang R. (2023). Composition characteristics, source and sensitivity to O_3_ formation of volatile organic compounds in Weihai. Environ. Prot. Sci..

[10] Li Q., Su G., Li C., Liu P., Zhao X., Zhang C., Sun X., Mu Y., Wu M., Wang Q. (2020). An investigation into the role of VOCs in SOA and ozone production in Beijing, China. Sci. Total Environ..

[11] Liu B., Liang D., Yang J., Dai Q., Bi X., Feng Y., Yuan J., Xiao Z., Zhang Y., Xu H. (2016). Characterization and source apportionment of volatile organic compounds based on 1-year of observational data in Tianjin, China. Environ. Pollut..

[12] Yurdakul S., Civan M., Kuntasal Ö., Doğan G., Pekey H., Tuncel G. (2018). Temporal variations of VOC concentrations in Bursa atmosphere. Atmos. Pollut. Res..

[13] Qian X., Shen H., Chen Z. (2019). Characterizing summer and winter carbonyl compounds in Beijing atmosphere. Atmos. Environ..

[14] Cheng Y., Lee S.C., Huang Y., Ho K.F., Ho S.S.H., Yau P.S., Louie P.K.K., Zhang R.J. (2014). Diurnal and seasonal trends of carbonyl compounds in roadside, urban, and suburban environment of Hong Kong. Atmos. Environ..

[15] Huang Y., Li X., Chen X., Wang W., Wang Y., Liu Z., Tang G. (2022). Low-molecular-weight carbonyl volatile organic compounds on the North China Plain. Atmos. Environ..

[16] Xiao Y., Hu M., Li X., Zong T., Xu N., Hu S., Zeng L., Chen S., Song Y., Guo S. (2022). Aqueous secondary organic aerosol formation attributed to phenols from biomass burning. Sci. Total Environ..

[17] Smith J.D., Kinney H., Anastasio C. (2015). Aqueous benzene-diols react with an organic triplet excited state and hydroxyl radical to form secondary organic aerosol. Phys. Chem. Chem. Phys..

[18] Crawford J., Faroon O., Llados F., Wilson J.D. (2008). Toxicological Profile for Phenol.

[19] MacFarlane S.M., Fisher J.A., Xu L., Wennberg P.O., Crounse J.D., Ball K., Zhai S., Bates K.H., Kim Y., Zhang Q. (2025). Sources, Sinks, and Oxidation Pathways of Phenolic Compounds in South Korea Constrained Using KORUS-AQ Airborne Observations. J. Geophys. Res. Atmos..

[20] Leuenberger C., Ligocki M.P., Pankow J.F. (1985). Trace organic compounds in rain. 4. Identities, concentrations, and scavenging mechanisms for phenols in urban air and rain. Environ. Sci. Technol..

[21] Belloli R., Barletta B., Bolzacchini E., Meinardi S., Orlandi M., Rindone B. (1999). Determination of toxic nitrophenols in the atmosphere by high-performance liquid chromatography. J. Chromatogr. A.

[22] Rubio M.A., Lissi E., Herrera N., Pérez V., Fuentes N. (2012). Phenol and nitrophenols in the air and dew waters of Santiago de Chile. Chemosphere.

[23] Lüttke J., Scheer V., Levsen K., Wünsch G., Neil Cape J., Hargreaves K.J., Storeton-West R.L., Acker K., Wieprecht W., Jones B. (1997). Occurrence and formation of nitrated phenols in and out of cloud. Atmos. Environ..

[24] Delhomme O., Morville S., Millet M. (2010). Seasonal and diurnal variations of atmospheric concentrations of phenols and nitrophenols measured in the Strasbourg area, France. Atmos. Pollut. Res..

[25] Smith J.D., Sio V., Yu L., Zhang Q., Anastasio C. (2014). Secondary Organic Aerosol Production from Aqueous Reactions of Atmospheric Phenols with an Organic Triplet Excited State. Environ. Sci. Technol..

[26] Li M., Wang X., Lu C., Li R., Zhang J., Dong S., Yang L., Xue L., Chen J., Wang W. (2020). Nitrated phenols and the phenolic precursors in the atmosphere in urban Jinan, China. Sci. Total Environ..

[27] Liang Y., Wang X., Dong S., Liu Z., Mu J., Lu C., Zhang J., Li M., Xue L., Wang W. (2020). Size distributions of nitrated phenols in winter at a coastal site in north China and the impacts from primary sources and secondary formation. Chemosphere.

[28] Xiao M., Wang M., Mentler B., Garmash O., Lamkaddam H., Molteni U., Simon M., Ahonen L., Amorim A., Baccarini A. (2025). Anthropogenic organic aerosol in Europe produced mainly through second-generation oxidation. Nat. Geosci..

[29] Volkamer R., Klotz B., Barnes I., Imamura T., Wirtz K., Washida N., Becker K.H., Platt U. (2002). OH-initiated oxidation of benzene. Phys. Chem. Chem. Phys..

[30] Olariu R.I., Klotz B., Barnes I., Becker K.H., Mocanu R. (2002). FT–IR study of the ring-retaining products from the reaction of OH radicals with phenol, o-, m-, and p-cresol. Atmos. Environ..

[31] Yan L.J., Bai Y.H., Zhao R.F., Li F., Xie K.C. (2015). Correlation between coal structure and release of the two organic compounds during pyrolysis. Fuel.

[32] Wang Y., Hu M., Wang Y., Zheng J., Shang D., Yang Y., Liu Y., Li X., Tang R., Zhu W. (2019). The formation of nitro-aromatic compounds under high NOx and anthropogenic VOC conditions in urban Beijing, China. Atmos. Chem. Phys..

[33] Liang Z., Li Y., Go B.R., Chan C.K. (2024). Complexities of Photosensitization in Atmospheric Particles. ACS EST Air.

[34] Bolzacchini E., Bruschi M., Hjorth J., Meinardi S., Orlandi M., Rindone B., Rosenbohm E. (2001). Gas-phase reaction of phenol with NO_3_. Environ. Sci. Technol..

[35] Cai M., Zhao Z., Li X., Wang G., Yang Y., Zhao W., Wang B., Yang H., Chen K., Ge S. (2024). Aqueous-Phase Photooxidation of Vanillin in the Presence of Nitrite: Characteristics, Products, and Mechanism. ACS EST Air.

[36] Atkinson R., Aschmann S.M., Arey J. (2002). Reactions of hydroxyl and nitrogen trioxide radicals with phenol, cresols, and 2-nitrophenol at 296 .+-. 2 K. Environ. Sci. Technol..

[37] McGillen M.R., Carter W.P.L., Mellouki A., Orlando J.J., Picquet-Varrault B., Wallington T.J. (2020). Database for the kinetics of the gas-phase atmospheric reactions of organic compounds. Earth Syst. Sci. Data.

[38] Yu J., Wu B., Peng C., Wentzell J., Wheeler M.J., Osagu J.O., Zhang X., Li L., Abbatt J.P.D., Liggio J. (2025). Multiphase OH Oxidation of Bisphenols: Chemical Transformation and Persistence in the Environment. Environ. Sci. Technol..

[39] Choi J., Jang M., Blau S. (2024). Dual roles of the inorganic aqueous phase on secondary organic aerosol growth from benzene and phenol. Atmos. Chem. Phys..

[40] Cheng X., Chen Q., Li Y., Huang G., Liu Y., Lu S., Zheng Y., Qiu W., Lu K., Qiu X. (2021). Secondary Production of Gaseous Nitrated Phenols in Polluted Urban Environments. Environ. Sci. Technol..

[41] Garmash O., Rissanen M.P., Pullinen I., Schmitt S., Kausiala O., Tillmann R., Zhao D., Percival C., Bannan T.J., Priestley M. (2020). Multi-generation OH oxidation as a source for highly oxygenated organic molecules from aromatics. Atmos. Chem. Phys..

[42] Bejan I., Schürmann A., Barnes I., Benter T. (2011). Kinetics of the gas-phase reactions of OH radicals with a series of trimethylphenols. Int. J. Chem. Kinet..

[43] Bejan I.G., Olariu R.I., Wiesen P. (2020). Secondary Organic Aerosol Formation from Nitrophenols Photolysis under Atmospheric Conditions. Atmosphere.

[44] An Z., Sun J., Han D., Mei Q., Wei B., Wang X., He M. (2019). Theoretical study on the mechanisms, kinetics and ecotoxicity assessment of OH-initiated reactions of guaiacol in atmosphere and wastewater. Sci. Total Environ..

[45] Couzo E., Lefer B., Stutz J., Yarwood G., Karamchandani P., Henderson B., Vizuete W. (2015). Impacts of heterogeneous HONO formation on radical sources and ozone chemistry in Houston, Texas. Atmos. Environ..

[46] Berndt T., Böge O. (2003). Gas-phase reaction of OH radicals with phenol. Phys. Chem. Chem. Phys..

[47] Xu C., Wang L. (2013). Atmospheric Oxidation Mechanism of Phenol Initiated by OH Radical. J. Phys. Chem. A.

[48] Mvula E., von Sonntag C. (2003). Ozonolysis of phenols in aqueous solution. Org. Biomol. Chem..

[49] Schwantes R.H., Schilling K.A., McVay R.C., Lignell H., Coggon M.M., Zhang X., Wennberg P.O., Seinfeld J.H. (2017). Formation of highly oxygenated low-volatility products from cresol oxidation. Atmos. Chem. Phys..

[50] Zaytsev A., Koss A.R., Breitenlechner M., Krechmer J.E., Nihill K.J., Lim C.Y., Rowe J.C., Cox J.L., Moss J., Roscioli J.R. (2019). Mechanistic study of the formation of ring-retaining and ring-opening products from the oxidation of aromatic compounds under urban atmospheric conditions. Atmos. Chem. Phys..

[51] Pillar-Little E.A., Camm R.C., Guzman M.I. (2014). Catechol Oxidation by Ozone and Hydroxyl Radicals at the Air–Water Interface. Environ. Sci. Technol..

[52] Ji Y., Zhao J., Terazono H., Misawa K., Levitt N.P., Li Y., Lin Y., Peng J., Wang Y., Duan L. (2017). Reassessing the atmospheric oxidation mechanism of toluene. Proc. Natl. Acad. Sci. USA.

[53] Butkovskaya N.I., Pouvesle N., Kukui A., Bras G.L. (2006). Mechanism of the OH-initiated oxidation of glycolaldehyde over the temperature range 233–296 K. J. Phys. Chem. A.

[54] Crutzen P.J., Lawrence M.G., Pöschl U. (1999). On the background photochemistry of tropospheric ozone. Tellus B Chem. Phys. Meteorol..

[55] Hofzumahaus A., Lefer B.L., Monks P.S., Hall S.R., Kylling A., Mayer B., Shetter R.E., Junkermann W., Bais A., Calvert J.G. (2004). Photolysis frequency of O_3_ to O(^1^D): Measurements and modeling during the International Photolysis Frequency Measurement and Modeling Intercomparison (IPMMI). J. Geophys. Res. Atmos..

[56] Jiang W., Yu L., Yee L., Chhabra P., Seinfeld J., Anastasio C., Zhang Q. (2024). Chemical Differences between Phenolic Secondary Organic Aerosol Formed through Gas-Phase and Aqueous-Phase Reactions. ACS Earth Space Chem..

[57] Hu J., Chen Z., Qin X., Dong P. (2022). Reversible and irreversible gas–particle partitioning of dicarbonyl compounds observed in the real atmosphere. Atmos. Chem. Phys..

[58] Chen Z., Chen K., Li X., Li R., Li Z., Xiao B., Wang G. (2026). Characterization, sources and reactivity of carbonyl volatile organic compounds in North China: Based on long-term observations. J. Environ. Sci..

[59] Li F., Zhou S., Du L., Zhao J., Hang J., Wang X. (2023). Aqueous-phase chemistry of atmospheric phenolic compounds: A critical review of laboratory studies. Sci. Total Environ..

[60] Jiang W., Niedek C., Anastasio C., Zhang Q. (2023). Photoaging of phenolic secondary organic aerosol in the aqueous phase: Evolution of chemical and optical properties and effects of oxidants. Atmos. Chem. Phys..

[61] Ferracci V., Heimann I., Abraham N.L., Pyle J.A., Archibald A.T. (2018). Global modelling of the total OH reactivity: Investigations on the “missing” OH sink and its atmospheric implications. Atmos. Chem. Phys..

[62] Atkinson R., Arey J. (2003). Atmospheric degradation of volatile organic compounds. Chem. Rev..

[63] Chen K., Gu X., Cai M., Zhao W., Wang B., Yang H., Liu X., Li X. (2025). Emission characteristics, environmental impacts and health risk assessment of volatile organic compounds from the typical chemical industry in China. J. Environ. Sci..

[64] Carter W.P.L. (2010). Development of the SAPRC-07 chemical mechanism. Atmos. Environ..

[65] Carter W.P.L. (1994). Development of Ozone Reactivity Scales for Volatile Organic-Compounds. J. Air Waste Manag. Assoc..

[66] Sahar G., Zhu H.W., Durbin T.D., Cocker D.R., Georgios K. (2022). The impact of hydrogenated vegetable oil (HVO) on the formation of secondary organic aerosol (SOA) from in-use heavy-duty diesel vehicles. Sci. Total Environ..

[67] Liu R., Chen J., Li G., Wang X., An T. (2019). Cutting down on the ozone and SOA formation as well as health risks of VOCs emitted from e-waste dismantlement by integration technique. J. Environ. Manag..

[68] Li J., Deng S., Li G., Lu Z., Song H., Gao J., Sun Z., Xu K. (2021). VOCs characteristics and their ozone and SOA formation potentials in autumn and winter at Weinan, China. Environ. Res..

[69] Grosjean D. (1992). In situ organic aerosol formation during a smog episode: Estimated production and chemical functionality. Atmos. Environ. Part A Gen. Top..

[70] Grosjean D., Seinfeld J.H. (1989). Parameterization of the formation potential of secondary organic aerosols. Atmos. Environ. (1967).

[71] Grosjean E., Grosjean D. (1999). The reaction of unsaturated aliphatic oxygenates with ozone. J. Atmos. Chem..

[72] Dechapanya W., Russell M., Allen D.T. (2004). Estimates of Anthropogenic Secondary Organic Aerosol Formation in Houston, Texas Special Issue of Aerosol Science and Technology on Findings from the Fine Particulate Matter Supersites Program. Aerosol Sci. Technol..

[73] Huang X.F., Zhang B., Xia S.Y., Han Y., Wang C., Yu G.H., Feng N. (2020). Sources of oxygenated volatile organic compounds (OVOCs) in urban atmospheres in North and South China. Environ. Pollut..

[74] Zhao Q., Bi J., Liu Q., Ling Z., Shen G., Chen F., Qiao Y., Li C., Ma Z. (2020). Sources of volatile organic compounds and policy implications for regional ozone pollution control in an urban location of Nanjing, East China. Atmos. Chem. Phys..

[75] Guo W., Yang Y., Chen Q., Zhu Y., Zhang Y., Zhang Y., Liu Y., Li G., Sun W., She J. (2022). Chemical reactivity of volatile organic compounds and their effects on ozone formation in a petrochemical industrial area of Lanzhou, Western China. Sci. Total Environ..

[76] Wang D., Zhou J., Han L., Tian W., Wang C., Li Y., Chen J. (2023). Source apportionment of VOCs and ozone formation potential and transport in Chengdu, China. Atmos. Pollut. Res..

[77] Zhang G., Zhou X., Sun Y., Han C., Xian J., Mu C., Xu W., Liang L. (2025). Oxygenated volatile organic compounds in Beijing: Characteristics, chemical reactivity, and source identification. Environ. Pollut..

[78] Wei Y., Jing X., Chen Y., Sun W., Zhang Y., Zhu R. (2024). Spatial-Temporal Characteristics, Source Apportionment, and Health Risks of Atmospheric Volatile Organic Compounds in China: A Comprehensive Review. Toxics.

[79] Ye C., Yuan B., Lin Y., Wang Z., Hu W., Li T., Chen W., Wu C., Wang C., Huang S. (2021). Chemical characterization of oxygenated organic compounds in the gas phase and particle phase using iodide CIMS with FIGAERO in urban air. Atmos. Chem. Phys..

[80] Liu Z., Zha F., Wang Y., Yuan B., Liu B., Tang G. (2023). Vertical evolution of the concentrations and sources of volatile organic compounds in the lower boundary layer in urban Beijing in summer. Chemosphere.

[81] Russell A., Milford J., Bergin M.S., McBride S., McNair L., Yang Y., Stockwell W.R., Croes B. (1995). Urban Ozone Control and Atmospheric Reactivity of Organic Gases. Science.

[82] Yang Y., Shao M., Keßel S., Li Y., Lu K., Lu S., Williams J., Zhang Y., Zeng L., Nölscher A.C. (2017). How the OH reactivity affects the ozone production efficiency: Case studies in Beijing and Heshan, China. Atmos. Chem. Phys..

[83] Whalley L.K., Stone D., Bandy B., Dunmore R., Hamilton J.F., Hopkins J., Lee J.D., Lewis A.C., Heard D.E. (2016). Atmospheric OH reactivity in central London: Observations, model predictions and estimates of in situ ozone production. Atmos. Chem. Phys..

[84] Lou S., Holland F., Rohrer F., Lu K., Bohn B., Brauers T., Chang C.C., Fuchs H., Häseler R., Kita K. (2010). Atmospheric OH reactivities in the Pearl River Delta—China in summer 2006: Measurement and model results. Atmos. Chem. Phys..

[85] Shirley T.R., Brune W.H., Ren X., Mao J., Lesher R., Cardenas B., Volkamer R., Molina L.T., Molina M.J., Lamb B. (2006). Atmospheric oxidation in the Mexico City Metropolitan Area (MCMA) during April 2003. Atmos. Chem. Phys..

[86] Ramasamy S., Nagai Y., Takeuchi N., Yamasaki S., Shoji K., Ida A., Jones C., Tsurumaru H., Suzuki Y., Yoshino A. (2018). Comprehensive measurements of atmospheric OH reactivity and trace species within a suburban forest near Tokyo during AQUAS-TAMA campaign. Atmos. Environ..

[87] Liu Y., Shao M., Kuster W.C., Goldan P.D., Li X., Lu S., Gouw J.A.d. (2009). Source Identification of Reactive Hydrocarbons and Oxygenated VOCs in the Summertime in Beijing. Environ. Sci. Technol..

[88] Yang Y., Wang Y., Zhou P., Yao D., Ji D., Sun J., Wang Y., Zhao S., Huang W., Yang S. (2020). Atmospheric reactivity and oxidation capacity during summer at a suburban site between Beijing and Tianjin. Atmos. Chem. Phys..

[89] Wei N., Zhao W., Zhang T., Wang J., Zhang C., Chen Y., Yu X., Lin W., Ye C., Fittschen C. (2025). Bridging the Gap Between Total Peroxyl Radical Observations and Models by Measuring OH Reactivity to Improve Estimates of Ozone Production: A Case Study From the 24th Winter Olympics Games. J. Geophys. Res. Atmos..

[90] Illmann N. (2025). A perspective on the reactions of organic peroxy radicals with HO_2_. Environ. Sci. Atmos..

[91] Whalley L.K., Slater E.J., Woodward-Massey R., Ye C., Lee J.D., Squires F., Hopkins J.R., Dunmore R.E., Shaw M., Hamilton J.F. (2021). Evaluating the sensitivity of radical chemistry and ozone formation to ambient VOCs and NOx in Beijing. Atmos. Chem. Phys..

[92] Chen L., Huang Y., Xue Y., Jia Z., Wang W. (2021). Atmospheric oxidation of 1-butene initiated by OH radical: Implications for ozone and nitrous acid formations. Atmos. Environ..

[93] Xiao F., Sun X., Li Z., Li X. (2020). Theoretical Study of Radical–Molecule Reactions with Negative Activation Energies in Combustion: Hydroxyl Radical Addition to Alkenes. ACS Omega.

[94] Zhang X., Luo J., Pan W., Xue Q., Liu X., Fu J., Zhang A., Jiang G. (2025). Implications of VOC oxidation in atmospheric chemistry: Development of a comprehensive AI model for predicting reaction rate constants. Atmos. Chem. Phys..

[95] Chen Z., Chen K., Yang Z., Yang F., Zhao W., Li X. (2026). Characterization of atmospheric volatile phenolic compounds in china: Based on long-term observations—Possibly seriously underestimated OVOCs. Atmos. Res..

[96] Wang W., Yuan B., Su H., Cheng Y., Qi J., Wang S., Song W., Wang X., Xue C., Ma C. (2024). A large role of missing volatile organic compound reactivity from anthropogenic emissions in ozone pollution regulation. Atmos. Chem. Phys..

[97] Wu R., Li J., Hao Y., Li Y., Zeng L., Xie S. (2016). Evolution process and sources of ambient volatile organic compounds during a severe haze event in Beijing, China. Sci. Total Environ..

[98] Yan Y., Peng L., Li R., Li Y., Li L., Bai H. (2017). Concentration, ozone formation potential and source analysis of volatile organic compounds (VOCs) in a thermal power station centralized area: A study in Shuozhou, China. Environ. Pollut..

[99] Ho K.F., Lee S.C., Guo H., Tsai W.Y. (2004). Seasonal and diurnal variations of volatile organic compounds (VOCs) in the atmosphere of Hong Kong. Sci. Total Environ..

[100] Barletta B., Meinardi S., Rowland F.S., Chan C.Y., Wang X.M., Zou S.C., Chan L.Y., Blake D.R. (2005). Volatile organic compounds in 43 Chinese cities. Atmos. Environ..

[101] Wu F., Yu Y., Sun J., Zhang J., Wang J., Tang G., Wang Y. (2016). Characteristics, source apportionment and reactivity of ambient volatile organic compounds at Dinghu Mountain in Guangdong Province, China. Sci. Total Environ..

[102] Ou J., Feng X., LIu Y., Gao Z., Yang Y., Zhang Z., Wang X., Zheng J. (2014). Source characteristics of VOCs emissions from vehicular exhaust in the Pearl River Delta region. Acta Sci. Circumstantiae.

[103] Pal R., Kim K., Hong Y., Jeon E. (2008). The pollution status of atmospheric carbonyls in a highly industrialized area. J. Hazard. Mater..

[104] Dörter M., Odabasi M., Yenisoy-Karakaş S. (2020). Source apportionment of biogenic and anthropogenic VOCs in Bolu plateau. Sci. Total Environ..

[105] Shen W., Mu Y., Wang B., Ai Z., Zhang L. (2017). Enhanced aerobic degradation of 4-chlorophenol with iron-nickel nanoparticles. Appl. Surf. Sci..

[106] Huang X., Zhao G., Liu M., Li F., Qiao J., Zhao S. (2012). Highly sensitive electrochemical determination of 1-naphthol based on high-index facet SnO_2_ modified electrode. Electrochim. Acta.

[107] Krugly E., Martuzevicius D., Tichonovas M., Jankunaite D., Rumskaite I., Sedlina J., Racys V., Baltrusaitis J. (2015). Decomposition of 2-naphthol in water using a non-thermal plasma reactor. Chem. Eng. J..

[108] Li G., Wei W., Shao X., Nie L., Wang H., Yan X., Zhang R. (2018). A comprehensive classification method for VOC emission sources to tackle air pollution based on VOC species reactivity and emission amounts. J. Environ. Sci..

[109] Zhang Z., Zhang Y., Wang X., Lü S., Huang Z., Huang X., Yang W., Wang Y., Zhang Q. (2016). Spatiotemporal patterns and source implications of aromatic hydrocarbons at six rural sites across China’s developed coastal regions. J. Geophys. Res. Atmos..

[110] Cui Y.Y., Liu S., Bai Z., Bian J., Li D., Fan K., McKeen S.A., Watts L.A., Ciciora S.J., Gao R.-S. (2018). Religious burning as a potential major source of atmospheric fine aerosols in summertime Lhasa on the Tibetan Plateau. Atmos. Environ..

[111] Zhang Y., You B., Shang Y., Bao Q., Zhang Y., Pang X., Guo L., Fu J., Chen W. (2024). Characteristics and ozone formation potentials of volatile organic compounds in a heavy industrial urban agglomeration of Northeast China. Air Qual. Atmos. Health.

[112] Lee B.-c., Yoon H., Lee B., Kim P., Moon H.-B., Kim Y. (2021). Occurrence of bisphenols and phthalates in indoor dust collected from Korean homes. J. Ind. Eng. Chem..

[113] Xiao Z., Yang X., Gu H., Hu J., Zhang T., Chen J., Pan X., Xiu G., Zhang W., Lin M. (2024). Characterization and sources of volatile organic compounds (VOCs) during 2022 summer ozone pollution control in Shanghai, China. Atmos. Environ..

[114] Hui L., Liu X., Tan Q., Feng M., An J., Qu Y., Zhang Y., Jiang M. (2018). Characteristics, source apportionment and contribution of VOCs to ozone formation in Wuhan, Central China. Atmos. Environ..

[115] Wu Y., Mo Z., Wu Q., Fan Y., Chen X., Li H., Lin H., Huang X., Tang H., Liao D. (2024). A Year-Long Measurement and Source Contributions of Volatile Organic Compounds in Nanning, South China. Atmosphere.

[116] Kong L., Zhou L., Chen D., Luo L., Xiao K., Chen Y., Liu H., Tan Q., Yang F. (2023). Atmospheric oxidation capacity and secondary pollutant formation potentials based on photochemical loss of VOCs in a megacity of the Sichuan Basin, China. Sci. Total Environ..

[117] Liu X., Lu J., Li W., Liu Z., Tong Y., Chen H., Yu J., Ding Y. (2021). Characterization, source apportionment, and assessment of volatile organic compounds in a typical urban area of southern Xinjiang, China. Air Qual. Atmos. Health.

[118] Lai Y.-J., Wang X.-W., Liu J.-F. (2022). Occurrence of trimethyl phosphate and triethyl phosphate in a municipal wastewater treatment plant and human urine. Environ. Pollut. Bioavailab..

[119] Fuentes-Ferragud E., Miralles P., Lopez A., Ibanez M., Coscolla C. (2023). Non-target screening and human risk assessment for adult and child populations of semi-volatile organic compounds in residential indoor dust in Spain. Chemosphere.

[120] Niu Y., Yan Y., Xing Y., Duan X., Yue K., Dong J., Hu D., Wang Y., Peng L. (2024). Analyzing ozone formation sensitivity in a typical industrial city in China: Implications for effective source control in the chemical transition regime. Sci. Total Environ..

[121] Wang T., Tao J., Li Z., Lu X., Liu Y., Zhang X., Wang B., Zhang D., Yin S. (2024). Characteristic, source apportionment and effect of photochemical loss of ambient VOCs in an emerging megacity of Central China. Atmos. Res..

[122] Guan Y., Wang L., Wang S., Zhang Y., Xiao J., Wang X., Duan E., Hou L.a. (2020). Temporal variations and source apportionment of volatile organic compounds at an urban site in Shijiazhuang, China. J. Environ. Sci..

[123] Ma Z., Liu C., Zhang C., Liu P., Ye C., Xue C., Zhao D., Sun J., Du Y., Chai F. (2019). The levels, sources and reactivity of volatile organic compounds in a typical urban area of Northeast China. J. Environ. Sci..

[124] Zou Y., Yan X.L., Flores R.M., Zhang L.Y., Yang S.P., Fan L.Y., Deng T., Deng X.J., Ye D.Q. (2023). Source apportionment and ozone formation mechanism of VOCs considering photochemical loss in Guangzhou, China. Sci. Total Environ..

[125] Magesh N.S., Tiwari A., Botsa S.M., da Lima Leitao T. (2021). Hazardous heavy metals in the pristine lacustrine systems of Antarctica: Insights from PMF model and ERA techniques. J. Hazard. Mater..

[126] Wen X.Y., Zhao W.T., Luo S.Z., Zhang Q., Wang Y.T., Ma J.J., Liu X.G. (2022). Pollution Characteristics and Source Apportionment of Atmospheric Volatile Organic Compounds in Summer in Yuncheng City. Huan Jing Ke Xue.

[127] Sun X.Y., Zhao M., Shen H.Q., Liu Y., Du M.Y., Zhang W.J., Xu H.Y., Fan G.L., Gong H.L., Li Q.S. (2022). Ozone Formation and Key VOCs of a Continuous Summertime O_3_ Pollution Event in Ji’nan. Huan Jing Ke Xue.

[128] Liu H., Tong J., Yang H., Liu Y., Ao C., Wang S. (2024). Comparative Study of VOCs Pollution Characteristics and Sources Analysis between Lanzhou Downtown Area and Xigu Refining Chemical Industrial Zone. Environ. Eng..

