# Supplementary material for: Undervalued Contribution of OVOCs to Atmospheric Activity: A Case Study in Beijing
Source: Toxics. 2026 Jan 14;14(1):77. doi: 10.3390/toxics14010077 (PMC12845771; doi:10.3390/toxics14010077)
Supplement: Supplementary file 1 [file toxics-14-00077-s001.zip › toxics-4051523-supplementary.pdf]

toxics

MDPI

Supplementary Data

Undervalued Contribution of OVOCs to Atmospheric Activity: A Case Study in Beijing

Kaitao Chen [1,2,†], Ziyan Chen [1,†], Fang Yang [1], Xingru Li [1] and Fangkun Wu [3,*]

[1] Analysis and Testing Center, Department of Chemistry, Capital Normal University, Beijing 100048, China; ckt0106@126.com (K.C.); zy.chen27@foxmail.com (Z.C.); fangyang1107@163.com (F.Y.); lixr@cnu.edu.cn (X.L.)
[2] Key Laboratory of Geographic Information Science of the Ministry of Education, School of Geographic Sciences, East China Normal University, Shanghai 200241, China
[3] State Key Laboratory of Atmospheric Boundary Layer Physics and Atmospheric Chemistry (LAPC), Institute of Atmospheric Physics, Chinese Academy of Sciences, Beijing 100029, China
* Correspondence: wfk@mail.iap.ac.cn
† These authors contributed equally to this work.

Supplementary materials

Text S1: Sample collection and analytical methods for carbonyl compounds.
Text S2: Establishment of standard curve for carbonyl compounds.
Text S3: Establishment of standard curve for volatile phenols.
Text S4: PMF model.
Table S1 HPLC mobile phase elution gradients.
Table S2 Standard curves and correlation coefficients for carbonyl compounds.
Table S3 Standard curves and correlation coefficients for volatile phenols.
Table S4: Method detection limit, precision and recovery rate of labeling for carbonyl compounds.
Table S5: Method detection limit (MDL), precision and recovery rate of labeling for volatile phenols.
Table S6: Atmospheric VOCs species fractions collected simultaneously.
Table S7: Source profiles of VOCs and their contribution percentages in 19 province and cities in China.
Fig. S1 Source profiles of resolved factors for the PMF model (gray bars represent mixing ratios, red dots represent percentages).
Fig. S2 Ternary diagram of benzene, toluene, and ethylbenzene.
Fig. S3 Chinese elevation map
Fig. S4 Diel profiles of six decomposed factors (a) and concentration diel profiles of key tracers of the Secondary generation source (b).

Text S1: Sample collection and analytical methods for carbonyl compounds.

1. Sample collection

Carbonyl compounds were referenced to the US Environmental Protection Agency (US EPA) TO-11A standard and China's HJ 683-2014 "Determination of Aldehydes and Ketones in Ambient Air by High Performance Liquid Chromatography", and samples were collected using a LIFE XP personal sampling pump (Beijing Bct Technology Ltd.) and a 2,4-dinitrophenylhydrazine (DNPH) sampling tube (CNWBOND DNPH-Silica, 350 mg, Amperexperiment) for sample collection, and a KI ozone removal column was connected to the front end of the DNPH sampling tube to prevent airborne O_3 from reacting with the DNPH derivatising agent and the derivatised hydrazone compounds. The sample flow rate was 800 mL/min and the sampling duration was 3 hours (8:00-11:00, 12:00-15:00 and 17:00-20:00). Upon completion of sampling, the DNPH sampling tubes were sealed, cooled and transported to the laboratory, where they were stored at 4 ℃ under refrigeration and analysis was completed in a timely manner. Excluding sampling failures due to power outages, rainy weather and other unforeseen events during the sampling process, a total of 42 valid samples and 3 blanks were collected in this study. Simultaneous sampling was carried out for volatile phenols.

2. Sample analysis

The sample pretreatment method for carbonyl compounds was acetonitrile reverse elution. A glass syringe was used to aspirate 5 mL of acetonitrile (HPLC grade, Fisher Chemical), and a 0.22 μm organic filter membrane was attached to the front end of the sampling tube, and the eluent was allowed to flow slowly into a 5 mL glass centrifuge tube, which was nitrogen-blown and volume determined to 1.5 mL, and then transferred to a brown liquid phase injection vial for measurement. After sample preparation, the samples were determined by high performance liquid chromatography with ultraviolet detector (HPLC-UV) (Thermo Fisher, UltiMate 3000), characterized by the retention time of the standards and quantified by the external standard method of chromatographic peak area. The chromatographic column was an Acclaim 120 C18 column (5 μm, 25 cm × 4.6 mm) and the temperature was set at 40 ℃. The sample volume was 20.0 mm. The injection volume was 20.0 μL, the UV detection wavelength was 360 nm (the detection wavelength for glyoxal and methylglyoxal was 420 nm) and the flow rate was 1.2 mL/min. The mobile phases were acetonitrile (HPLC grade, 99.95%, Fisher Chemical) and addition of 0.1% formic acid (HPLC grade, 99%, DIKMA) in ultra-pure water, the elution gradient is shown in **Table S1** below; a further 5 min equilibration method was set to change 100% acetonitrile linearly to 60% / 40% (acetonitrile / ultra-pure water) before running the next sample.

Text S2: Establishment of standard curve for carbonyl compounds.

The standard use solution (ϱ = 10 μg/mL) was used to formulate the standard series with concentrations of 0.1, 0.2, 0.5, 1.0, 2.0, 5.0 and 10.0 μg/mL. The standard curve of methylglyoxal was configured with the concentration gradient of 0.01, 0.02, 0.05, 0.1, 0.2, 0.5 and 1.0 μg/mL.20.0 μL of the standard series were taken in order from low to high concentration and injected into the HPLC. The calibration curve was plotted with the chromatographic response value as the vertical coordinate and the carbonyl compound concentration (μg/mL) as the horizontal coordinate, as shown in **Table S2**.

Text S3: Establishment of standard curve for volatile phenols.

Measure 0, 50, 100, 200, 500, 1000 μL of the standard use solution (ϱ=100 mg/L) into a 10 ml volumetric flask and mix well with methanol. The standard series with

concentrations of 0, 0.5, 1.0, 2.0, 5.0 and 10.0 mg/L were prepared. The 10.0 μL standard series were measured sequentially from low concentration to high concentration, injected into the HPLC and measured according to the reference chromatographic conditions, and the calibration curve was plotted with the chromatographic response value as the vertical coordinate and the concentration of phenolic compounds (mg/L) as the horizontal coordinate. The standard curve and the correlation coefficient of the phenolic compounds are presented in **Table S3** below. The correlation coefficient of the calibration curve was greater than or equal to 0.999.

Text S4: PMF model.

For the purpose of being able to identify and evaluate each resolved factor to make its physical meaning sensible the sample statistical matrix is first divided into two matrices of factor contributions (G) and and factor profiles (F) with non-negative restrictions. The EPA PMF 5.0 model was used in this study to analyze the main sources of phenols. The basic algorithm is as follows [1]:

$$x_{ij} = \sum_{k=1}^{p} g_{ik} f_{kj} + e_{ij} \quad (1)$$

where x_{ij} is the concentration of substance j in sample i; p is the number of factors, i.e., the number of sources; g_{ik} is the factor spectrum, i.e., it represents the relative contribution of sample i in the kth factor (source); f_{kj} is the source contribution, i.e., the amount of substance j in the k-factor composition spectrum; and e_{ij} is the residuals of substance j of sample i in the PMF calculation process. The sample concentration and its related uncertainty are needed by the PMF so as to weight the points. The function Q is minimized by the PMF model:

$$Q = \sum_{i=1}^{n} \sum_{j=1}^{m} \left[\frac{x_{ij} - \sum_{k=1}^{p} g_{ik} f_{kj}}{u_{ij}} \right] \quad (2)$$

The EPA PMF 5.0 model's parsing process deleted missing data. Substance concentrations below the MDL are used to calculate the uncertainty using the fixed fraction of MDL (Eq. 3). Concentrations above the MDL, the uncertainty is calculated based on the concentration and MDL fraction (Eq. 4).

$$Unc = \frac{5}{6} \times MDL \quad (3)$$

$$Unc = \sqrt{(ErrorFraction \times concentration)^2 + (0.5 \times MDL)^2} \quad (4)$$

Table S1. HPLC mobile phase elution gradients.

Compounds	Time / min	Ultra-pure water / %	Acetonitrile / %
Volatile phenols	0	70	30
	7.5	45	55
	9.5	20	80
	11.0	0	100
Carbonyl compounds	0	40	60
	7	30	70
	12	40	60
	16	32	68
	19	32	68
	24	20	80
	25	0	100

Table S2. Standard curves and correlation coefficients for carbonyl compounds.

**Compounds**	**Standard Curves**	**Correlation Coefficients**
Formaldehyde	y = 11.387x + 0.7495	0.9996
Acetaldehyde	y = 8.5547x + 0.3474	0.9997
Acrolein	y = 7.9748x + 0.1951	0.9998
Acetone	y = 6.4015x + 0.2647	0.9998
Propanal	y = 6.6035x + 0.1987	0.9998
Butenal	y = 5.9469x + 0.2121	0.9998
Isobutenal	y = 1.6164x - 0.0785	0.9999
Butyraldehyde	y = 5.4610x + 0.0974	0.9998
Benzaldehyde	y = 4.0142x + 0.1073	0.9997
Isovaleraldehyde	y = 4.6089x + 0.0169	0.9999
Valeraldehyde	y = 4.5417x + 0.0400	1.0000
O-methylbenzaldehyde	y = 3.4646x + 0.0539	1.0000
M/p-methylbenzaldehyde	y = 3.4494x + 0.1928	0.9999
Hexanal	y = 4.0011x + 0.0552	1.0000
2,5-Dimethylbenzaldehyde	y = 2.9719x + 0.0663	0.9999
Glyoxal	y = 1.2479x - 0.0396	0.9999
Methylglyoxal	y = 11.8830x - 0.0040	0.9971

Table S3. Standard curves and correlation coefficients for volatile phenols.

**Compounds**	**Standard Curves**	**Correlation Coefficients**
Resorcinol	y = 0.0794x - 0.0097	0.9993
Phenol	y = 0.0599x - 0.007	0.9991
M/p-cresol	y = 0.0765x - 0.0124	0.9994
O-cresol	y = 0.0648x - 0.0061	0.9995
2,4-Dinitrophenol	y = 0.3656x - 0.0337	0.9995
4-Chlorophenol	y = 0.0673x - 0.0077	0.9994
2-Naphthol	y = 0.1862x - 0.0157	0.9994
2,6-Dimethylphenol	y = 0.0434x - 0.0039	0.9996
1-Naphthol	y = 0.2056x - 0.0147	0.9994
2,4-Dichlorophenol	y = 0.0734x - 0.0193	0.9993

Table S4. Method detection limit, precision and recovery rate of labeling for carbonyl compounds.

**Compounds**	**MDL（ppbv）**	**Instrument precision RSD_i（%）**	**Method Precision RSD_m（%）**	**Recovery rate of labeling （%）**
Formaldehyde	0.0074	0.94	0.40	95.21
Acetaldehyde	0.0044	0.74	0.45	95.08
Acrolein	0.0033	0.95	0.67	98.15
Acetone	0.0077	0.51	1.71	95.31
Propanal	0.0067	1.24	0.49	106.41
Butenal	0.0044	0.76	0.50	96.72
Isobutenal	0.0080	1.69	0.29	97.32
Butyraldehyde	0.0042	1.43	0.23	100.97
Benzaldehyde	0.0074	0.93	0.32	105.04
Isovaleraldehyde	0.0086	1.87	1.23	90.09
Valeraldehyde	0.0079	1.59	1.54	101.17
O-methylbenzaldehyde	0.0025	2.00	1.13	111.70
M/p-methylbenzaldehyde	0.0053	1.55	0.42	106.61
Hexanal	0.0031	0.87	0.40	88.30
2,5-Dimethylbenzaldehyde	0.0070	0.72	0.30	96.54
Glyoxal	0.0089	0.66	0.54	64.12
Methylglyoxal	0.0104	2.80	3.63	62.17

Table S5. LOD, precision and recovery rate of labeling for volatile phenols.

**Compounds**	**LOD（ppbv）**	**Instrument precision RSD_i（%）**	**Method Precision RSD_m（%）**	**Recovery rate of labeling（%）**
Resorcinol	0.4855	5.22	6.46	110.41
Phenol	0.6880	6.39	5.65	113.12
M/p-cresol	0.6724	4.24	3.84	104.45
O-cresol	0.6203	7.54	5.96	109.97
2,4-Dinitrophenol	0.1538	2.14	2.99	112.49
4-Chlorophenol	0.3977	4.36	7.31	96.65
2-Naphthol	0.2519	3.77	6.90	104.57
2,6-Dimethylphenol	0.4967	7.73	8.01	96.95
1-Naphthol	0.2233	3.12	3.80	97.73
2,4-Dichlorophenol	0.2944	5.47	7.51	126.22

Table S6. Atmospheric VOCs species fractions collected simultaneously.

**Type and quantity of VOCs**	**Specific components**
Alkanes (28)	Ethane, propane, isobutane, butane, isopentane, n-pentane, cyclopentane, 2,2-dimethylbutane, 2,3-dimethylbutane, 2-methylpentane, 3-methylpentane, n-hexane, 2,4-dimethylpentane, methylcyclopentane, 2-methylhexane, cyclohexane, 2,2,4-trimethylpentane, 3-methylhexane, n-heptane, methylcyclohexane, 2,3,4-trimethylpentane, 2-methylheptane, 3-methylheptane, octane, nonane, decane, n-undecane, n-dodecane
Alkenes (9)	Propylene, 1-butene, trans-2-butene, cis-2-butene, 1-pentene, cis-2-pentene, trans-2-pentene, isoprene, hexene
Aromatics (15)	Benzene, toluene, ethylbenzene, p/m-xylene, styrene, o-xylene, isopropylbenzene, n-propylbenzene, p/m-ethyltoluene, 1,2,4-trimethylbenzene, o-ethyltoluene, 1,2,3-trimethylbenzene, 1,3,5-trimethylbenzene, m-diethylbenzene, p-diethylbenzene
Carbonyls (17)	Formaldehyde, acetaldehyde, acrolein, acetone, propanal, butenal, isobutenal, butyraldehyde, benzaldehyde, isovaleraldehyde, valeraldehyde, o-methylbenzaldehyde, m/p-methylbenzaldehyde, hexanal, 2,5-dimethylbenzaldehyde, glyoxal, methylglyoxal
Volatile phenols (10)	Resorcinol、phenol、o-cresol、m/p-cresol、2,4-dinitrophenol、4-chlorophenol、1-naphthol、2-naphthol、2,6-dimethylphenol、2,4-dichlorophenol

Table S7. Source profiles of VOCs and their contribution percentages in 1 province and 18 cities in China.

**Province & Cities**	**Time**	**Vehicle exhaust**	**Fuel evaporation**	**Solvent use and organic chemical**	**Biogenic**	**Petrochemical industry**	**NG/LPG**	**Industrial process**	**Combustion**	**Automobile exhaust**	**Secondary generation**	**Others**	**References**
Beijing	2023	27.5	13.7	31.7	15.6	11.5	—	—	—	—	—	—	This study
Shanghai	2022	17.0	16.0	17.7	—	16.6	17.5	15.2	—	—	—	—	[2]
Nanjing	2018-2020	39.0	16.0	8.0	—	—	18.0	—	19.0	—	—	—	[3]
Shijiazhuang	2018-2020	12.2	6.8	11.6	—	26.2	6.5	15.2	21.5	—	—	—	[4]
Lhasa	2021	12.0	—	7.0	—	—	13.0	—	29.0	—	39.0	—	[5]
Weihai	2022	56.0	—	7.1	5.0	—	—	6.8	25.1	—	—	—	[6]
Tianjin	2015	12.7	—	4.1	6.2	—	19.5	17.8	6.3	27.7	—	5.7	[7]
Yuncheng	2020	33.1	—	11.9	8.2	—	17.3	29.5	—	—	—	—	[8]
Jinan	2019	35.4	22.4	15.0	8.9	—	—	—	18.3	—	—	—	[9]
Wuhan	2017	22.1	10.5	10.8	6.0	—	24.3	16.1	10.2	—	—	—	[10]
Lanzhou	2021	35.8	30.5	16.6	—	—	—	9.5	7.6	—	—	—	[11]
Zhengzhou	2019	30.1	7.4	11.4	5.3	—	15.9	22.0	8.0	—	—	—	[12]
Nanning	2020-2021	33.0	—	16.0	5.0	—	19.0	10.0	17.0	—	—	—	[13]
Chengdu	2019	35.5	—	20.3	4.6	—	25.0	14.6	—	—	—	—	[14]
Changzhi	2021	42.9	—	14.0	7.6	—	—	15.1	20.3	—	—	—	[15]
Tumushuke	2020	26.1	18.4	—	11.9	—	—	12.8	30.9	—	—	—	[16]
Shenyang	2017	36.2	6.3	10.6	14.3	9.9	5.8	—	16.9	—	—	—	[17]

Guang-zhou	2021	27.0	17.0	11.0	14.0	—	—	31.0	—	—	—	—	[18]
Jilin prov-ince	2020	4.6	—	2.7	—	—	—	70.6	21.4	—	—	0.6	[19]

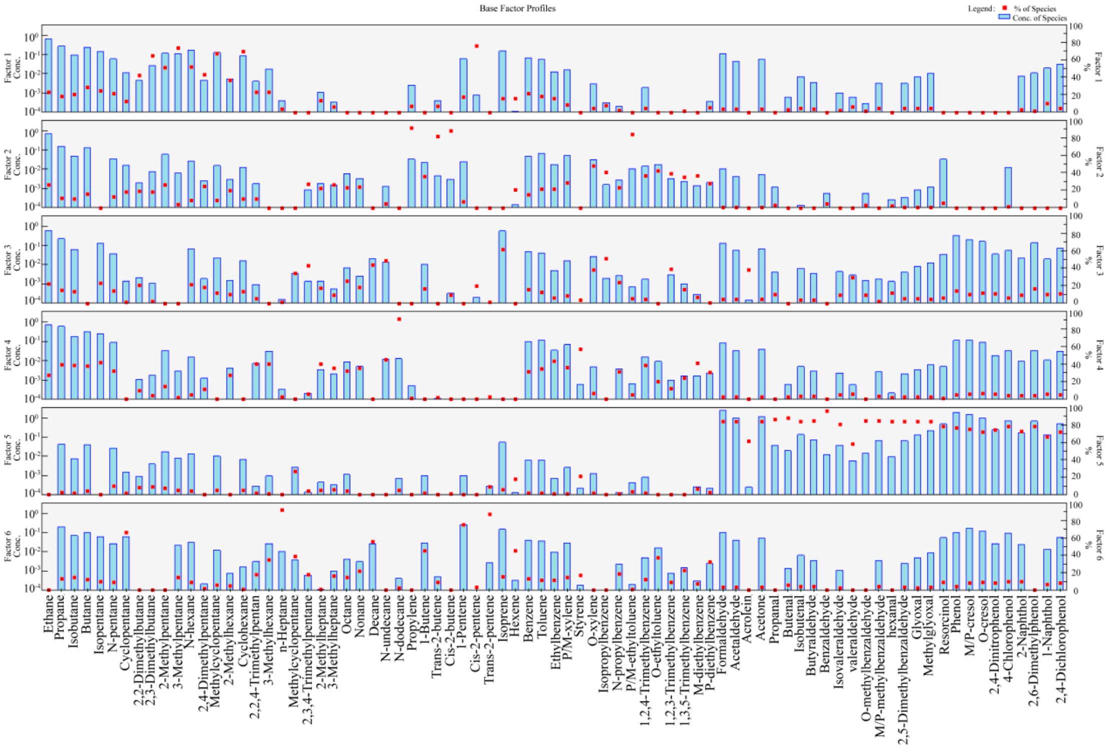

Figure S1. Source profiles of resolved factors for the PMF model (gray bars represent mixing ratios, red dots represent percentages).

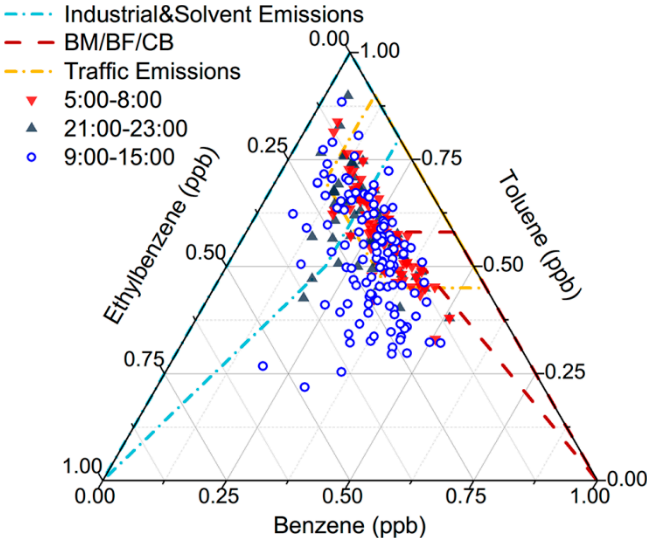

Figure S2. Ternary diagram of benzene, toluene, and ethylbenzene. Plot suggests the combined contribution to aromatics from vehicle emissions, solvent usage, and biomass burning. BM/BF/CB indicates biomass/biofuel/coal burning. The wireframes include more than 90% of the scatter col-lected from references [20].

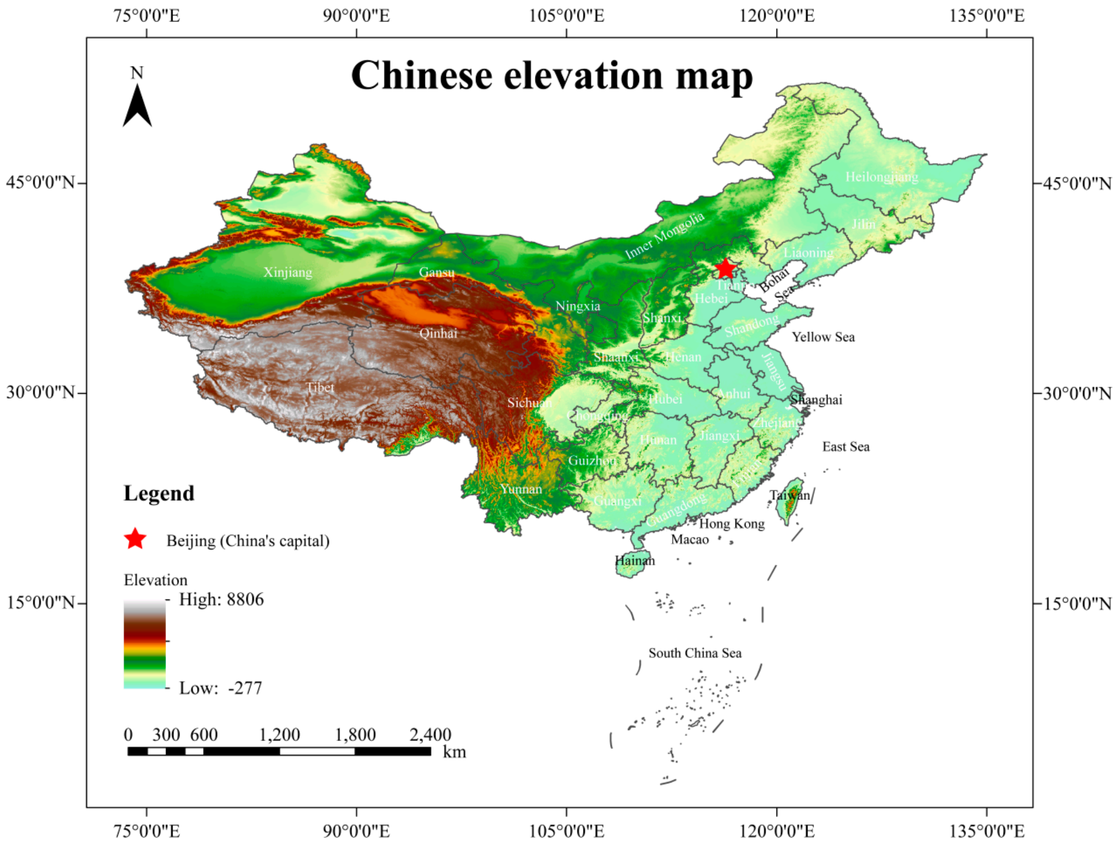

Figure S3. Chinese elevation map.

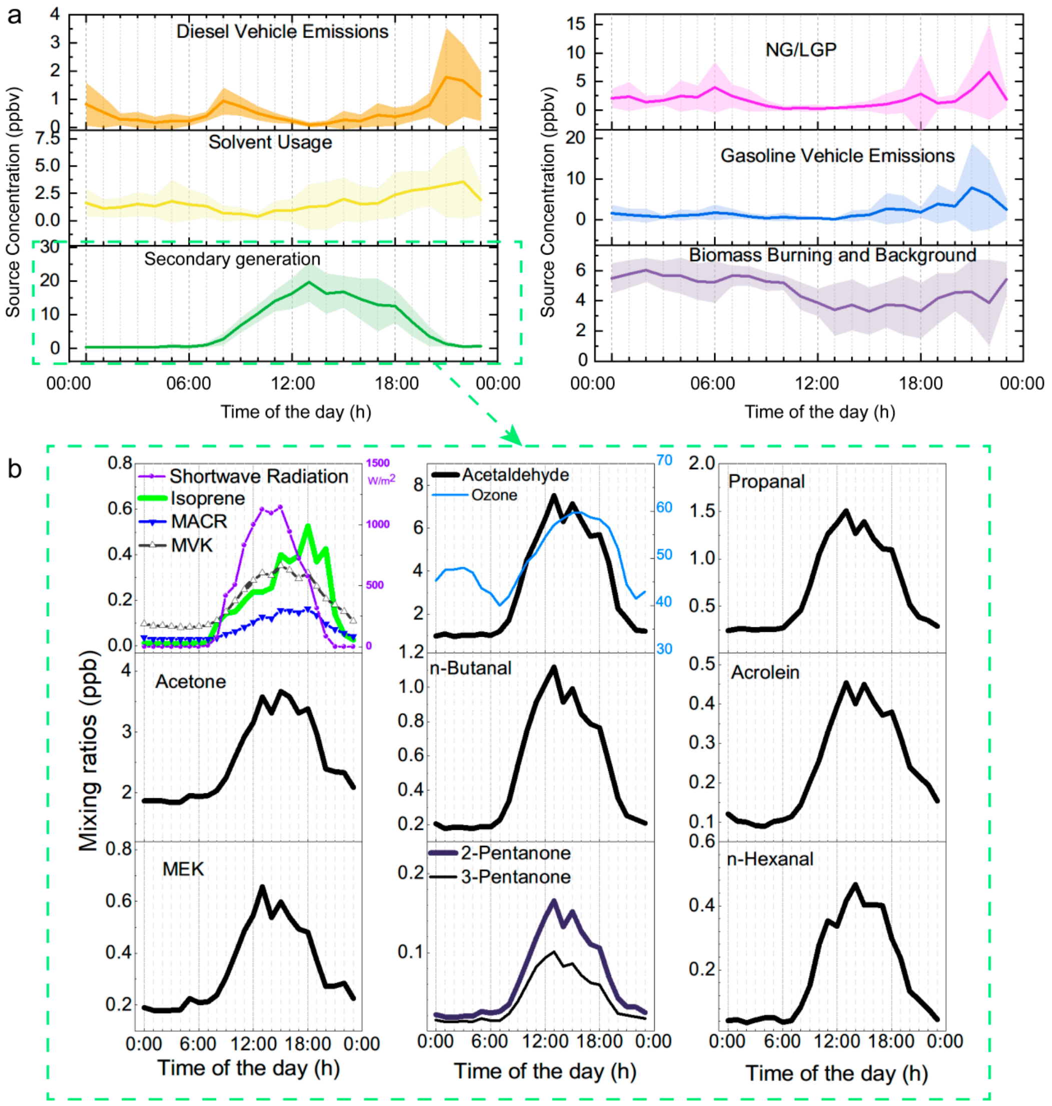

Figure S4. Diel profiles of six decomposed factors (a) and concentration diel profiles of key tracers of the Secondary generation source (b) [5].

References

1. Magesh, N.S.; Tiwari, A.; Botsa, S.M.; da Lima Leitao, T. Hazardous heavy metals in the pristine lacustrine systems of Antarctica: Insights from PMF model and ERA techniques. *J Hazard Mater* **2021**, *412*, 125263, doi:10.1016/j.jhazmat.2021.125263.
2. Xiao, Z.; Yang, X.; Gu, H.; Hu, J.; Zhang, T.; Chen, J.; Pan, X.; Xiu, G.; Zhang, W.; Lin, M. Characterization and sources of volatile organic compounds (VOCs) during 2022 summer ozone pollution control in Shanghai, China. *Atmospheric Environment* **2024**, *327*, 120464, doi:10.1016/j.atmosenv.2024.120464.
3. Mozaffar, A.; Zhang, Y.-L.; Lin, Y.-C.; Xie, F.; Fan, M.-Y.; Cao, F. Measurement report: High contributions of halocarbon and aromatic compounds to atmospheric volatile organic compounds in an industrial area. *Atmospheric Chemistry and Physics* **2021**, *21*, 18087-18099, doi:10.5194/acp-21-18087-2021.
4. Guan, Y.; Wang, L.; Wang, S.; Zhang, Y.; Xiao, J.; Wang, X.; Duan, E.; Hou, L. Temporal variations and source apportionment of volatile organic compounds at an urban site in Shijiazhuang, China. *J Environ Sci (China)* **2020**, *97*, 25-34, doi:10.1016/j.jes.2020.04.022.
5. Ye, C.; Guo, S.; Lin, W.; Tian, F.; Wang, J.; Zhang, C.; Chi, S.; Chen, Y.; Zhang, Y.; Zeng, L.; et al. Measurement report: Source apportionment and environmental impacts of volatile organic compounds (VOCs) in Lhasa, a highland city in China. *Atmospheric Chemistry and Physics* **2023**, *23*, 10383-10397, doi:10.5194/acp-23-10383-2023.
6. Zhang, X.; Wang, L.; Jin, X.; Zhang, R. Composition characteristics, source and sensitivity to O_3 formation of volatile organic compounds in Weihai. *Environmental Protection Science* **2023**, *49*, 130-137, doi:10.16803/j.cnki.issn.1004-6216.202306035.

7. Liu, B.; Liang, D.; Yang, J.; Dai, Q.; Bi, X.; Feng, Y.; Yuan, J.; Xiao, Z.; Zhang, Y.; Xu, H. Characterization and source apportionment of volatile organic compounds based on 1-year of observational data in Tianjin, China. *Environ Pollut* **2016**, *218*, 757-769, doi:10.1016/j.envpol.2016.07.072.
8. Wen, X.Y.; Zhao, W.T.; Luo, S.Z.; Zhang, Q.; Wang, Y.T.; Ma, J.J.; Liu, X.G. [Pollution Characteristics and Source Apportionment of Atmospheric Volatile Organic Compounds in Summer in Yuncheng City]. *Huan Jing Ke Xue* **2022**, *43*, 2979-2986, doi:10.13227/j.hjkx.202109134.
9. Sun, X.Y.; Zhao, M.; Shen, H.Q.; Liu, Y.; Du, M.Y.; Zhang, W.J.; Xu, H.Y.; Fan, G.L.; Gong, H.L.; Li, Q.S.; et al. Ozone Formation and Key VOCs of a Continuous Summertime O3 Pollution Event in Ji'nan. *Huan Jing Ke Xue* **2022**, *43*, 686-695, doi:10.13227/j.hjkx.202106068.
10. Hui, L.; Liu, X.; Tan, Q.; Feng, M.; An, J.; Qu, Y.; Zhang, Y.; Jiang, M. Characteristics, source apportionment and contribution of VOCs to ozone formation in Wuhan, Central China. *Atmospheric Environment* **2018**, *192*, 55-71, doi:10.1016/j.atmosenv.2018.08.042.
11. Liu, H.; Tong, J.; Yang, H.; Liu, Y.; Ao, C.; Wang, S. Comparative Study of VOCs Pollution Characteristics and Sources Analysis between Lanzhou Downtown Area and Xigu Refining Chemical Industrial Zone. *Environmental Engineering* **2024**, *42*, 139-147, doi:10. 13205 /j. hjgc. 202404017.
12. Wang, T.; Tao, J.; Li, Z.; Lu, X.; Liu, Y.; Zhang, X.; Wang, B.; Zhang, D.; Yin, S. Characteristic, source apportionment and effect of photochemical loss of ambient VOCs in an emerging megacity of Central China. *Atmospheric Research* **2024**, *305*, 107429, doi:10.1016/j.atmosres.2024.107429.
13. Wu, Y.; Mo, Z.; Wu, Q.; Fan, Y.; Chen, X.; Li, H.; Lin, H.; Huang, X.; Tang, H.; Liao, D.; et al. A Year-Long Measurement and Source Contributions of Volatile Organic Compounds in Nanning, South China. *Atmosphere* **2024**, *15*, 560, doi:10.3390/atmos15050560.
14. Kong, L.; Zhou, L.; Chen, D.; Luo, L.; Xiao, K.; Chen, Y.; Liu, H.; Tan, Q.; Yang, F. Atmospheric oxidation capacity and secondary pollutant formation potentials based on photochemical loss of VOCs in a megacity of the Sichuan Basin, China. *Sci Total Environ* **2023**, *901*, 166259, doi:10.1016/j.scitotenv.2023.166259.
15. Niu, Y.; Yan, Y.; Xing, Y.; Duan, X.; Yue, K.; Dong, J.; Hu, D.; Wang, Y.; Peng, L. Analyzing ozone formation sensitivity in a typical industrial city in China: Implications for effective source control in the chemical transition regime. *Sci Total Environ* **2024**, *919*, 170559, doi:10.1016/j.scitotenv.2024.170559.
16. Liu, X.; Lu, J.; Li, W.; Liu, Z.; Tong, Y.; Chen, H.; Yu, J.; Ding, Y. Characterization, source apportionment, and assessment of volatile organic compounds in a typical urban area of southern Xinjiang, China. *Air Quality, Atmosphere & Health* **2021**, *15*, 785-797, doi:10.1007/s11869-021-01133-4.
17. Ma, Z.; Liu, C.; Zhang, C.; Liu, P.; Ye, C.; Xue, C.; Zhao, D.; Sun, J.; Du, Y.; Chai, F.; et al. The levels, sources and reactivity of volatile organic compounds in a typical urban area of Northeast China. *J Environ Sci (China)* **2019**, *79*, 121-134, doi:10.1016/j.jes.2018.11.015.
18. Zou, Y.; Yan, X.L.; Flores, R.M.; Zhang, L.Y.; Yang, S.P.; Fan, L.Y.; Deng, T.; Deng, X.J.; Ye, D.Q. Source apportionment and ozone formation mechanism of VOCs considering photochemical loss in Guangzhou, China. *Sci Total Environ* **2023**, *903*, 166191, doi:10.1016/j.scitotenv.2023.166191.
19. Zhang, Y.; You, B.; Shang, Y.; Bao, Q.; Zhang, Y.; Pang, X.; Guo, L.; Fu, J.; Chen, W. Characteristics and ozone formation potentials of volatile organic compounds in a heavy industrial urban agglomeration of Northeast China. *Air Quality, Atmosphere & Health* **2024**, *17*, 2235-2246, doi:10.1007/s11869-024-01569-4.
20. Zhang, Z.; Zhang, Y.; Wang, X.; Lü, S.; Huang, Z.; Huang, X.; Yang, W.; Wang, Y.; Zhang, Q. Spatiotemporal patterns and source implications of aromatic hydrocarbons at six rural sites across China's developed coastal regions. *Journal of Geophysical Research: Atmospheres* **2016**, *121*, 6669-6687, doi:10.1002/2016jd025115.
